# The Structural Basis for Processing of Unnatural Base Pairs by DNA Polymerases

**DOI:** 10.1002/chem.201903525

**Published:** 2020-01-21

**Authors:** Andreas Marx, Karin Betz

**Affiliations:** ^1^ Department of Chemistry Konstanz Research School Chemical Biology University of Konstanz Universitätsstrasse 10 78464 Konstanz Germany

**Keywords:** DNA, DNA polymerase structures, KlenTaq DNA polymerase, nucleobases, unnatural base pairs (UBPs)

## Abstract

Unnatural base pairs (UBPs) greatly increase the diversity of DNA and RNA, furthering their broad range of molecular biological and biotechnological approaches. Different candidates have been developed whereby alternative hydrogen‐bonding patterns and hydrophobic and packing interactions have turned out to be the most promising base‐pairing concepts to date. The key in many applications is the highly efficient and selective acceptance of artificial base pairs by DNA polymerases, which enables amplification of the modified DNA. In this Review, computational as well as experimental studies that were performed to characterize the pairing behavior of UBPs in free duplex DNA or bound to the active site of KlenTaq DNA polymerase are highlighted. The structural studies, on the one hand, elucidate how base pairs lacking hydrogen bonds are accepted by these enzymes and, on the other hand, highlight the influence of one or several consecutive UBPs on the structure of a DNA double helix. Understanding these concepts facilitates optimization of future UBPs for the manifold fields of applications.

## Graphical abstract

**Hydrogen bonding or rather more hydrophobic?** Two different pairing concepts are the basis for the currently most promising unnatural base pair (UBP) candidates. This review mainly focusses on structural studies investigating UBP pairings in free duplex DNA or the active site of KlenTaq DNA polymerase, but also highlights the general mechanisms for the acceptance of these unnatural substrates by DNA polymerases.

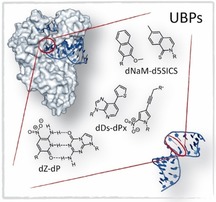

## 1 Introduction to Unnatural Base Pairs

**Figure 1:**
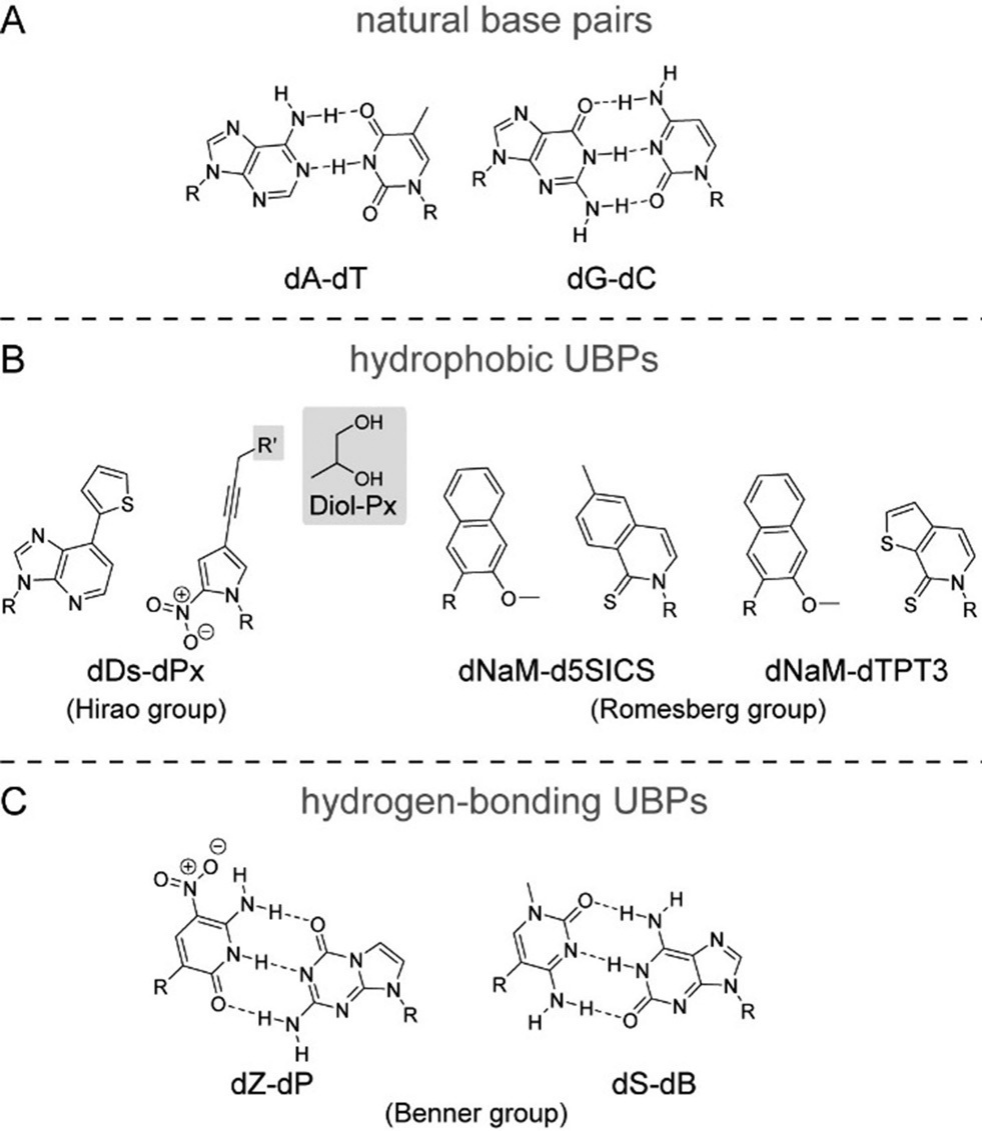
Chemical structures of A) natural base pairs and B,C) UBPs (R=2**′**‐deoxyribose or 2′‐deoxyribose‐5′‐triphosphate). B) Members of the family of hydrophobic UBPs of the Hirao group (left) and the Romesberg group (right). **dPx** can carry different modifications at position R′ (gray box). In this review, we discuss the **dPx** carrying a diol functionality. C) Two pairs from the Benner group, which are characterized by an alternative hydrogen‐bonding pattern compared with the natural pairs.

Genetic information in all living organisms is encoded in DNA, which consists of nucleotides with four different nucleobases that form nucleobase pairs. Adenine pairs with thymine (or uracil in RNA) through two hydrogen bonds and cytosine pairs with guanine through three hydrogen bonds (Figure 1 A). Decades ago, the plan emerged to design synthetic nucleotides that can form additional base pairs, so‐called artificial or unnatural base pairs (UBPs).^1^ The benefits of having a third base pair are diverse. As UBPs are structurally different from the natural pairs (differences can range from minimal to large), a clear gain is the increased chemical and structural diversity in DNA and RNA strands that can be created if it consists of six instead of four building blocks. Increased diversity is, for example, useful in the search for affinity binders like aptamers. Including an UBP in SELEX (systematic evolution of ligands by exponential enrichment) processes can be used to generate aptamers that bind to proteins and cells, as has already successfully been demonstrated.^2^, ^3^, ^4^, ^5^, ^6^, ^7^ Apart from generating diversity in DNA and RNA, a third base pair can be used to incorporate non‐proteinogenic amino acids into a polypeptide chain by ribosome‐based translation. Generation of proteins containing unnatural amino acids by the use of UBPs has already been realized in vitro^8^, ^9^ but would be even more useful in vivo and a first success in this field has already been achieved.^10^ One ultimate aim of synthetic biology is the generation of a semisynthetic organism (SSO) in which the artificial base pair is stably included during growth and reproduction. A future practical application of such an SSO would be the production of new proteins with therapeutic or diagnostic value, which include non‐natural amino acids at specific sites. Artificial base pairs can also be used for site‐specific post amplification labeling of DNA,^11^, ^12^ for example, to identify DNA lesions.^13^ Furthermore, Benner and co‐workers showed, for example, the beneficial contribution of their artificial pairs in multiplexed polymerase chain reaction (PCR),^14^ diagnostic of different viral RNA sequences in a complex environment,^15^ and the synthesis of large DNA constructs from short fragments.^16^

To be applicable in the above‐mentioned approaches, the UBP candidate needs to fulfill several properties. The UBP needs to be fully orthogonal to the natural pairs and efficiently and selectively replicated by DNA polymerases (during multiple cycles of PCR) and translated to RNA by RNA polymerases. Thereby, the pairing partners should be inserted into DNA with an error rate per base pair (also termed fidelity) at least as low as 10^−3^,^17^ meaning one error in 1000 incorporation reactions. For comparison, natural DNA is replicated with fidelities of up to 10^−5^ to 10^−6^ when using a DNA polymerase with an associated 3′–5′ exonuclease activity.^17^ One error in 1000 reactions would require a selectivity of at least 99.9 % per replication step. A 99.9 % selectivity in turn leads to 97 % retention of the UBP after 30 cycles of PCR (0.999^30^=0.97) and only 90 % retention after 100 cycles of PCR. Even though this degree of selectivity is sufficient for a number of applications (e.g., in the use of primers containing UBPs in nested PCR or use in diagnostics),^13^, ^14^ for others, where high amplification of the DNA or plasmid containing the UBP is performed and loss of the UBP is critical (e.g., if implemented in an SSO that should produce proteins containing an unnatural amino acid),^9^ a selectivity truly approaching that of natural pairs is crucial.

In this review, we feature different UBPs with the main focus on their acceptance by DNA polymerases and structural studies, investigating the base pairs in free duplex DNA and in the active site of KlenTaq DNA polymerase (Klenow Fragment of DNA polymerase I of *Thermus aquaticus*). We use the abbreviations UB nucleosides (dN), and UB nucleotides (dNMP for the monophosphate, dNTP for the triphosphate) in the following.

## 2 Different UBPs and their Acceptance by DNA Polymerases

Chiefly, three different groups headed by Benner, Romesberg, and Hirao have most significantly advanced the development of UBPs in the past few decades and all three groups have developed different candidate molecular scaffolds, which are well replicated by DNA polymerases (Figure 1 B, C). In this review, we only introduce the currently most successful and investigated pairs developed by these research groups. Thereby, we differentiate between the two families: hydrogen‐bonding UBPs (including the candidates from the Benner lab) and hydrophobic, non‐hydrogen‐bonding UBPs (comprising the most recent pairs developed in the Hirao and Romesberg labs). A detailed history of the development of UBPs is described elsewhere.^18^, ^19^, ^20^, ^21^, ^22^, ^23^ Furthermore, the numerous and diverse applications of the well‐replicated UBPs in the creation of DNA aptamers and an SSO with a six‐letter alphabet, but also other in vitro applications, are described in the following reviews.^22^, ^24^, ^25^, ^26^

### 2.1 Hydrogen‐bonding UBPs

Based on orthogonal hydrogen‐bonding patterns, the Benner lab developed a fully Artificially Expanded Genetic Information System (AEGIS) including 12 nucleotides that in total form six specific nucleobase pairs. All pairs have a different, distinct arrangement of hydrogen‐bond donor and acceptor groups, form three hydrogen bonds, and retain Watson–Crick geometries.^18^, ^27^ The most prominent members of the AEGIS system are the nucleobases 2‐amino‐imidazo[1,2‐*a*]‐1,3,5‐triazin‐4(8 *H*)one (shortly termed **P**) and 6‐amino‐5‐nitro‐2(1 *H*)‐pyridone (shortly termed **Z**), which form a **P**–**Z** base pair through three hydrogen bonds^28^ (Figure 1 C). The **dP**–**dZ** pair is replicated by diverse DNA polymerases of the A‐ and B‐family, albeit with lower efficiency compared with the natural counterparts.^29^ In PCR reactions using Taq (family A), Vent (*exo*‐), and DeepVent (*exo*‐) (both family B) DNA polymerases, the fidelity (or selectivity) per round is reported to be 94.4, 97.5, and 97.5 %, respectively. A more recent protocol runs with retention of one **dP**–**dZ** pair in an amplified DNA strand of 99.2 % per theoretical PCR cycle with standard triphosphate concentrations and even 99.8 % under optimized triphosphate concentrations.^30^ The study by Yang et al. revealed that the highest retention of the **dP**–**dZ** pair is reached at a pH of 7.8–8.0 by using Vent (*exo*‐) or DeepVent (*exo*‐) but with the drawback that natural **dC**–**dG** pairs are likely converted to **dP**–**dZ** pairs. For an optimal overall fidelity (low misincorporation of unnatural opposite natural nucleotides plus high retention of the unnatural nucleotides), Taq DNA polymerase appeared to be better.^29^ Several consecutive (up to four) **dP**–**dZ** pairs can be enzymatically incorporated into a DNA strand before the incorporation stops. Furthermore, DNA templates containing up to four consecutive **dP–dZ** pairs can be PCR amplified by Taq and Phusion DNA polymerases.^30^

Owing to the rather low incorporation efficiency, directed evolution of Taq DNA polymerase was performed to improve the enzyme properties. The generation of a KlenTaq DNA polymerase mutant (M444V, P527A, D551E, and E832V) increased the incorporation efficiency of **dZMP** opposite **dP** (judged by primer extension experiments).^31^, ^32^ The reverse process, incorporation of **dPMP** opposite a templating **dZ**, however, was inefficient. A major drawback of the **dP**–**dZ** pair in general is the mispairing with natural nucleotides (mainly misincorporation of **dGMP** opposite deprotonated **dZ)**.^30^, ^33^

### 2.2 Non‐hydrogen‐bonding, hydrophobic UBPs

A different strategy to develop artificial base pairs was followed by the Hirao and Romesberg groups. Both groups decided to investigate base pairs that structurally differ from the natural base pairs and that pair through hydrophobic and packing forces rather than hydrogen bonds. This approach was inspired by the work of Kool and co‐workers who showed that hydrogen bonds are not necessarily needed to form a base pair that can efficiently and selectively be replicated by DNA polymerases.^34^, ^35^ The Hirao group thereby focused on the concept of shape complementarity by combining one smaller and one larger scaffold like a natural pyrimidine–purine pair. The Romesberg group relied on structures with little to no homology to the natural counterparts (Figure 1 B).

The most prominent base pair from the Hirao group is the pair formed between 7‐(2‐thienyl)imidazo[4,5‐*b*]pyridine (**Ds**) and 2‐nitro‐4‐propynylpyrrole (**Px**) (Figure 1 B).^36^ This pair can efficiently and selectively be replicated by DNA polymerases and is successfully used in different applications, for example, in the generation of aptamers.^2^ The **Px** base can carry different functional groups at the propynyl linker such as amino, diol, and aromatic groups (see Figure 1 in ref. 36) or azide, ethynyl, and biotin (see Figure 1 in ref. 37). Several of these **dPx** nucleotides can easily be modified further (before and after insertion into DNA) with even large functional groups, which is a powerful tool for the generation of site‐specifically modified DNA.^37^ As the **diol‐dPx** (shown in Figure 1 B, gray box) was shown to be the best pairing partner of **dDs** in PCR amplification,^36^ we used it in our structural studies together with the Hirao group and refer to the **diol‐dPx** as just **dPx** throughout this review.

The Hirao pair **dDs**–**dPx** is most efficiently replicated by family B DNA polymerases. Members of the A‐family (Taq and TITANIUM Taq DNA polymerases, which have intrinsically no 3′–5′ exonuclease activity) showed much lower selectivity for the **dDs**–**dPx** pairing in PCR amplification with **dDs**‐containing templates.^36^ In an optimized protocol for PCR amplification of DNA containing **dDs** and **dPx**, the DeepVent (*exo*+) DNA polymerase (B‐family) is used.^36^, ^37^ The fidelities reached in these experiments (dependent on template sequence and modification at **dPx**) are 99.96 up to >99.97 % per doubling event. Although **dDs**–**dPx** is efficiently replicated within various DNA sequences, there are still some sequences preferred over others (for details see ref. 38). PCR amplification of DNA containing two **dDs** bases separated by 4, 6, 9, or 12 natural bases showed that at least six natural bases inserted between two **dDs** bases are needed to exhibit high amplification efficiency under the tested conditions.^38^

**Figure 2:**
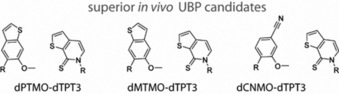
Base pairs that show improved retention in the environment of a semisynthetic organism (R=2′‐deoxyribose or 2′‐deoxyribose‐5′‐triphosphate).

In several rounds of screening and optimization based on structure–activity relationship data, the Romesberg group developed the base pair between 2,6‐dimethyl‐2 *H*‐isoquiniline‐1‐thione (**d5SICS**) and 2‐methoxy‐3‐methylnaphthalene (**dNaM**; Figure 1 B). The UBP **dNaM**–**d5SICS** was intensively studied and was the first artificial base pair to be replicated by the endogenous replication machinery in a plasmid in *E. coli* cells.^39^ The **dNaM**–**d5SICS** pair is most efficiently amplified by OneTaq, a mixture of the A‐family Taq DNA polymerase and the B‐family Deep Vent (*exo*+) DNA Polymerase.^40^ Depending on the template sequence, the remarkably high amplification fidelities per doubling of 99.66 to >99.98 % are reached.^40^ As PCR amplification with exonuclease‐deficient DNA polymerases proceeded with higher efficiency than when using exonuclease‐proficient enzymes (with standard concentrations of natural substrates) but exonuclease activity is needed to reach high fidelity, a mixture of two enzymes was found to yield the best results. Further optimization of the pair led to the, to date, most efficiently in vitro replicated pair **dNaM**–**dTPT3** (fidelity per doubling in replication: >99.98 %^12^), which was first used in the creation of an SSO that not only stores^41^ but also retrieves increased information.^10^ For the realization of an SSO, in vivo screening of base pair candidates led to the pairs **dMTMO**–**dTPT3**, **dPTMO**–**dTPT3**,^42^ and **dCNMO**–**dTPT3** 43 (Figure 2), which all show increased retention in an SSO compared with the **dNaM**–**dTPT3** pair. The fact that these pairs show increased replication proficiency, meaning higher retention rates in vivo but not in vitro, emphasizes the importance of the way of evaluating candidates. An important factor contributing to the different results in vitro and in vivo might be the different uptake of the substrates into the cell, or stability within the cell, the different DNA polymerases (e.g., *E. coli* Pol III and/or Pol II) that replicate DNA in the SSO,^43^ and the presence of other components in the in vivo replisome (e.g., the β‐clamp processivity factor or DNA repair mechanisms).^44^

### 2.3 Further artificial base pairs

Apart from the hydrogen‐bonding and hydrophobic UBPs just mentioned, new base‐pairing concepts have emerged. Size‐expanded base pairs, also termed benzo‐expanded DNA or xDNA^45^, ^46^ and base pairs with four instead of three or two hydrogen bonds^47^ have been introduced, but fidelities and efficiencies in polymerase reactions are currently low.^17^ Additionally, metal‐mediated base pairs that consist of two ligand‐type nucleobases connected through a central metal ion have been developed.^48^ Through its coordination, the metal ion stably crosslinks two strands and therefore these pairs are interesting in DNA nanotechnology.^49^, ^50^ Furthermore, DNA containing such pairs function as metal ion sensors and are, for example, used for the detection of Hg^2+^ in specimens.^48^, ^50^

## 3 Structure of Hydrophobic UBPs: The Hirao and Romesberg Pairs

It is remarkable that such high amplification efficiencies and fidelities are reached with artificial base pairs that significantly differ in shape compared with the natural base pairs and only rely on hydrophobic and packing forces. Understanding the structure of the hydrophobic UBPs themselves and their influence on the DNA structure in solution or their processing by enzymes is key to understanding the molecular basis of these processes and might enable optimizing candidates for different applications. In the following, we review structural data gained from experimental and computational studies on hydrophobic UBPs either as free pairs, in duplex DNA, or in the active site of a DNA polymerase.

The structures of hydrophobic artificial base pairs have been studied in different ways and contexts. **DNaM**–**d5SICS** and related base pairs from the Romesberg group were investigated as isolated pairs by computational methods and in free duplex DNA by means of computational and experimental methods. Further, the structures of **dNaM**–**d5SICS** and **dDs**–**dPx** in complexes with KlenTaq DNA polymerase were studied by using X‐ray crystallography in our group in collaboration with the Hirao and Romesberg groups.

### 3.1 Computational studies

DFT (dispersion‐corrected density functional theory) calculations of free nucleobases (sugar and phosphate moieties omitted) revealed that **NaM**–**5SICS**, **NaM**–**TPT3**, and related pairs favor a “slipped parallel stacked dimer arrangement”^51^, ^52^ with the nucleobases positioned on top of each other rather than forming a Watson–Crick‐type planar structure. The interplanar distance of the stacking bases is 3.3 to 3.5 Å and the exemplary center‐to‐center distance of **NaM**–**5SICS** is 3.6 Å.^51^ Negi et al. found similar distorted parallel geometries that enable π–π stacking for a number of hydrophobic pairs from the Romesberg group (including **NaM**–**5SICS**) but contradicting results were found for the pair **NaM**–**TPT3**.^53^ The nucleobases in **NaM**–**TPT3** do not stack but pair in a highly bent structure, which may in part be stabilized by a weak interaction between the sulfur of **TPT3** and the methoxy group of **NaM**.

### 3.2 Computational studies of structures within duplex DNA

**Figure 3:**
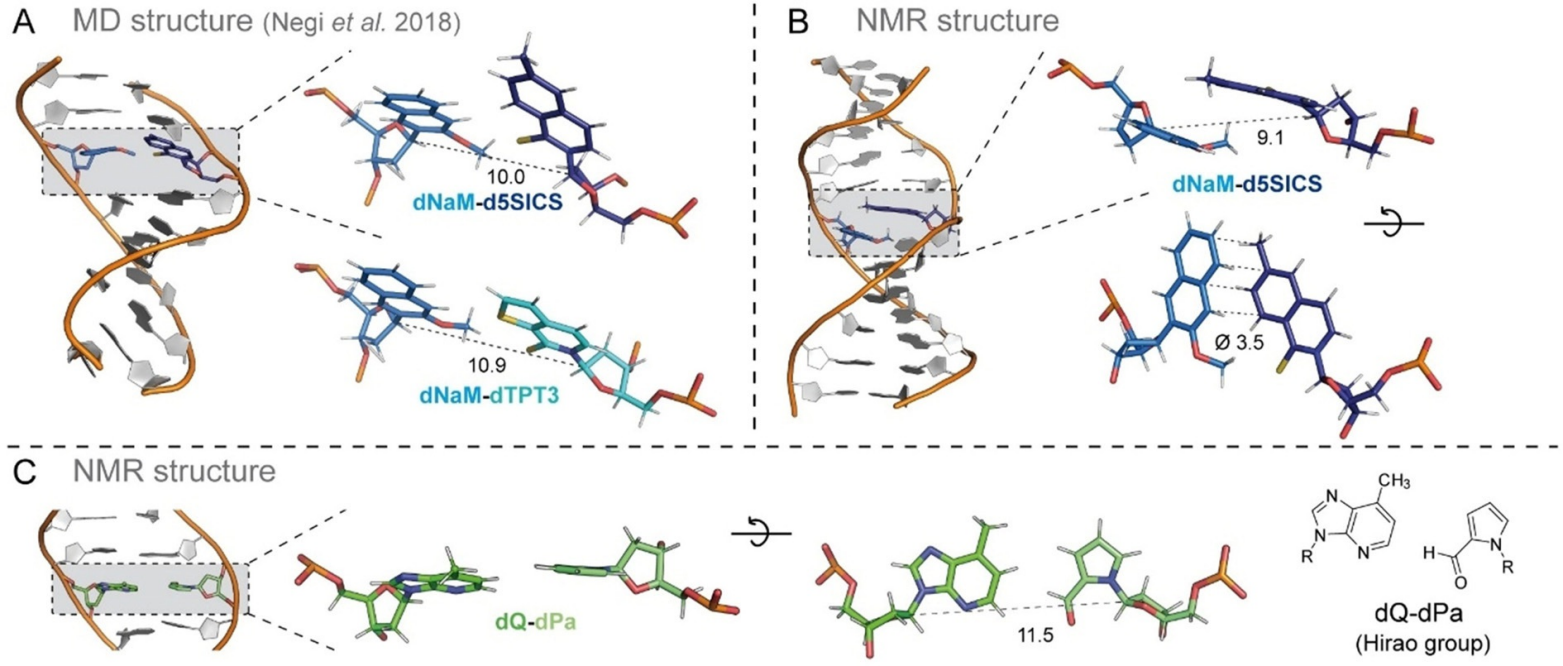
A,B) Comparison of hydrophobic UBP structures in free duplex DNA in A) a molecular modeling study^51^ and B) an NMR study.^53^, ^54^ The studied DNA duplexes containing a **dNaM**–**d5SICS** pair are shown on the left side with a close‐up of the base pair on the right side. **dNaM**, **d5SICS**, and **dTPT3** are shown in marine, dark blue, and cyan, respectively. A) The pairing of **dNaM**–**dTPT3** investigated in the same study is shown in addition. B) A second orientation of **dNaM**–**d5SICS** is shown to better visualize the stacking. Distances between the C1′ atoms of the ribose moieties and the average distance of the edges between **dNaM** and **d5SICS** in the NMR structure are given in Å. C) Structure of an NMR study investigating the **dQ**–**dPa** pair, a precursor of the **dDs**–**dPx** pair discussed in this review. The **dQ**–**dPa** pair in an edge‐to‐edge‐like manner with a slightly larger C1′−C1′ distance compared with the natural pairs in the strand (average distance of eleven natural pairs: 10.5 Å).

In contrast to the results from free nucleobases, classical molecular dynamics simulations show that when positioned within a DNA double strand (11‐mer), the **dNaM**–**d5SICS** pair forms a natural‐like planar structure with a C1′−C1′ distance of 10.7 Å.^51^ In this orientation, π‐stacking with natural bases above and below can be maximized. In this study, it was concluded that the stability of the UBPs arises from the rather strong dispersion interactions between the planar **d5SICS** and **dNaM** nucleobases and their neighboring stacked natural bases (intrastrand interactions) rather than the interactions within the UBP (interstrand). A similar result was found by molecular dynamics simulations by Negi et al., which shows that **d5SICS**–**dMMO2** (a precursor of **d5SICS**–**dNaM**) and **d5SICS**–**dNaM** (C1′−C1′ distance of 10.0 Å) adopt a nearly planar geometry and **dTPT3**–**dNaM** (C1′−C1′ distance of 10.9 Å) takes a completely planar geometry within DNA^53^ (Figure 3 A). Thereby, **dTPT3**–**dNaM** shows the least perturbations of the DNA double helix geometry. This result is consistent with the higher replication fidelity and efficiency of **dTPT3**–**dNaM** compared with many other hydrophobic UBPs.^12^

In another computational study, published by Galindo‐Murillo et al., a 13‐mer DNA strand containing one, three, or five **dNaM**–**d5SICS** base pairs was investigated.^54^ In the presence of one artificial pair, the group observed—similar to Jahiruddin et al. and Negi et al.—a rather planar orientation of the base pair in the most populated structure within a 10 μs simulation. This structure has a C1′−C1′ distance for the UBP of 11.4 Å, which is similar to natural pairs, however, other base pair and backbone geometry parameters differ greatly. DFT computations of a trimer with the **dNaM**–**d5SICS** in the middle again yield a slightly different result. The **dNaM**–**d5SICS** pair still adopts a somehow edge‐to‐edge structure with a C1′−C1′ distance of 10.8 Å but with a propeller angle of −13°, which significantly deviates from being planar. In the same study, molecular dynamics simulation results show that embedding more than two **dNaM**–**d5SICS** pairs within a short DNA strand (13‐mer), the structure of the double helix is heavily disturbed until it collapses (with five unnatural pairs).^54^

All in all, computational studies indicate stacking arrangements for isolated hydrophobic artificial base pairs but rather edge‐to‐edge oriented nucleobases with different extents of distortion when embedded within short sequences of natural DNA (**5SICS**–**FEMO** being an exception, for details see ref. 53). The different geometries of the UBPs found by computational methods in DNA and for isolated pairs emphasize the importance of interactions with neighboring (natural) nucleotides on the structure. The computational results (within short DNA sequences) support the use of hydrophobic artificial base pairs as they would not significantly hamper the stability and geometry of double helical DNA at least as long as only one UBP is embedded within natural nucleotides.^51^, ^52^, ^53^ The highly planar structure of **dNaM**–**dTPT3** that only weakly disturbs the overall DNA double helix correlates well with the high efficiency and fidelity of the pair in PCR. This fact renders the computational studies useful in screening for even better‐performing artificial base pair candidates.

### 3.3 NMR studies

Several years before the computational studies were performed, NMR studies revealed that the hydrophobic artificial base pair **dNaM**–**d5SICS** 55 and the related **dMMO2**–**d5SICS**,^56^ do not pair edge‐to‐edge like the hydrogen‐bonding natural pairs but adopt a partially intercalating structure in free duplex DNA (Figure 3 B). In detail, in the studied 12‐mer DNA duplex containing **dNaM**–**d5SICS** in the center of the duplex, the edges of the two nucleobases lie on top of each other with an average distance of 3.5 Å. In this state, the stacking interactions between the pairing partners seem to be maximized. The internucleotide distance between the C1′ atoms of the 2′‐deoxyribose moieties is 9.1 Å, which is significantly shorter compared with natural base pairs (usually around 10.4–10.5 Å for A–T and G–C pairs^57^). Interstrand intercalation has also been observed for other hydrophobic nucleobase analogs, for example, biphenyl and bipyridyl nucleotides,^58^, ^59^, ^60^ the self‐pair **PICS**–**PICS**,^61^ and aromatic chromophores.^62^, ^63^ Thus, intercalation seems to be a general feature of large aromatic nucleobase analogs, which is consistent with the important role of hydrophobicity and dispersion interactions.^22^

For the **dDs**–**dPx** pair, no structural information in free duplex DNA is available and it is thus not known which pairing geometry is adopted. A precursor of **dDs**–**dPx**, the pair **dQ**–**dPa** (Figure 3 C), however, was structurally studied by NMR spectroscopy within a 12‐mer DNA duplex.^64^ In this structure, the **dQ**–**dPa** pair forms a geometry similar to a Watson–Crick base pair although with some minor variations (bases are tilted with respect to each other, enlarged C1′−C1′ distance) and higher structural flexibility indicated by the broad NMR signals. Compared with **dQ**, **dDs** contains an additional thienyl moiety and dPx exhibits a nitro instead of the aldehyde group in dPa as well as an additional propynyl moiety. The main structures of these two pairs, however, are the same. Thus, it is likely that **dDs**–**dPx** can pair in a similar way as observed for **dQ**–**dPa** in an edge‐to‐edge, planar manner, closely resembling the geometry of a Watson–Crick base pair.

Taken together, the results of the NMR study with **dNaM**–**d5SICS** do not match the computational results that were obtained later (described in section 3.2). The discrepancy between an intercalating structure of **dNaM**–**d5SICS** and **dMMO2**–**d5SICS** within a DNA strand in the NMR studies and a more planar orientation of **dNaM**–**d5SICS** (and related pairs) in DNA in simulation studies needs to be further evaluated. It would furthermore be interesting to see if the **dDs**–**dPx** pair from the Hirao group, for which such studies were not made, likewise behaves differently in structural and computational studies.

The intercalating structure of **dNaM**–**d5SICS** was somehow surprising considering the proficient acceptance of the pair by DNA polymerases. This circumstance raised the question of how DNA polymerases deal with the artificial pair(s) on a molecular level and motivated structural studies.

### 3.4 Structure of UBPs in the active site of KlenTaq DNA polymerase

**Figure 4:**
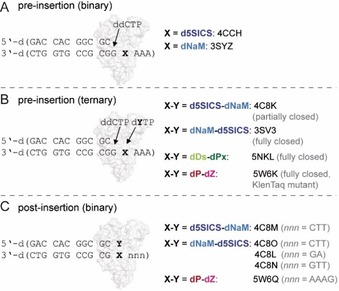
Crystallographic complexes trapped with KlenTaq WT or a KlenTaq mutant and UBPs in different states. The sequences of the primer/template duplexes used for crystallization are shown. Addition of ddCTP in A) and B) leads to **ddCMP** insertion and termination of the primer. PDB codes of the respective structures are given.

To shine light into the mechanisms of UBP recognition by DNA polymerases, we, together with the Romesberg and Hirao groups, decided to investigate hydrophobic artificial base pairs in the active site of the structurally and functionally well‐characterized KlenTaq DNA polymerase. For the enzymatic incorporation of artificial nucleotides into DNA, a template containing an artificial nucleotide at the templating position as well as a cognate substrate triphosphate have to be bound and recognized as a “correct” pair in the active site of a DNA polymerase. For the two hydrophobic artificial base pairs **dNaM**–**d5SICS** and **dDs**–**dPx**, several crystal structures with KlenTaq were solved. Our study in total resulted in eight crystal structures in four different reaction states of the enzyme with components of the **dNaM**–**d5SICS** pair whereas for the Hirao pair **dDs**–**dPx** one structure is available. An overview of all structures with KlenTaq is given in Figure 4 together with the protein database (PDB) codes. Based on the KlenTaq complexes with the **dNaM**–**d5SICS** pair and previously obtained functional and structural data,^56^, ^65^, ^66^, ^67^, ^68^ a mechanism of replication for hydrophobic artificial base pairs was proposed,^69^ which probably also holds true—at least in some aspects—for other similar or less hydrophobic artificial base pairs lacking hydrogen bonds. In this section, we introduce the obtained crystal structures containing hydrophobic unnatural nucleotides and compare them with the analogous natural binary complexes with a **dG** or **dT** at the templating position (termed KlenTaq_dG_ and KlenTaq_dT_, respectively) and the natural ternary complex with a **dG–dCTP** pair in the insertion site (termed KlenTaq_dG–dCTP_).

The general crystallization strategy for binary and ternary complexes is the following: the KlenTaq DNA polymerase is purified and mixed with a previously annealed primer/template complex (for sequences of duplexes, see Figure 4). For the pre‐insertion complexes, a terminator dideoxy nucleotide is added, which after insertion terminates the primer owing to the lack of the 3′‐OH group. Binary crystals are grown and either measured or soaked with the respective substrates to obtain ternary crystal structures. Soaking conditions differed for the three substrates (for details, see refs. 55, 69, 70).

**Figure 5:**
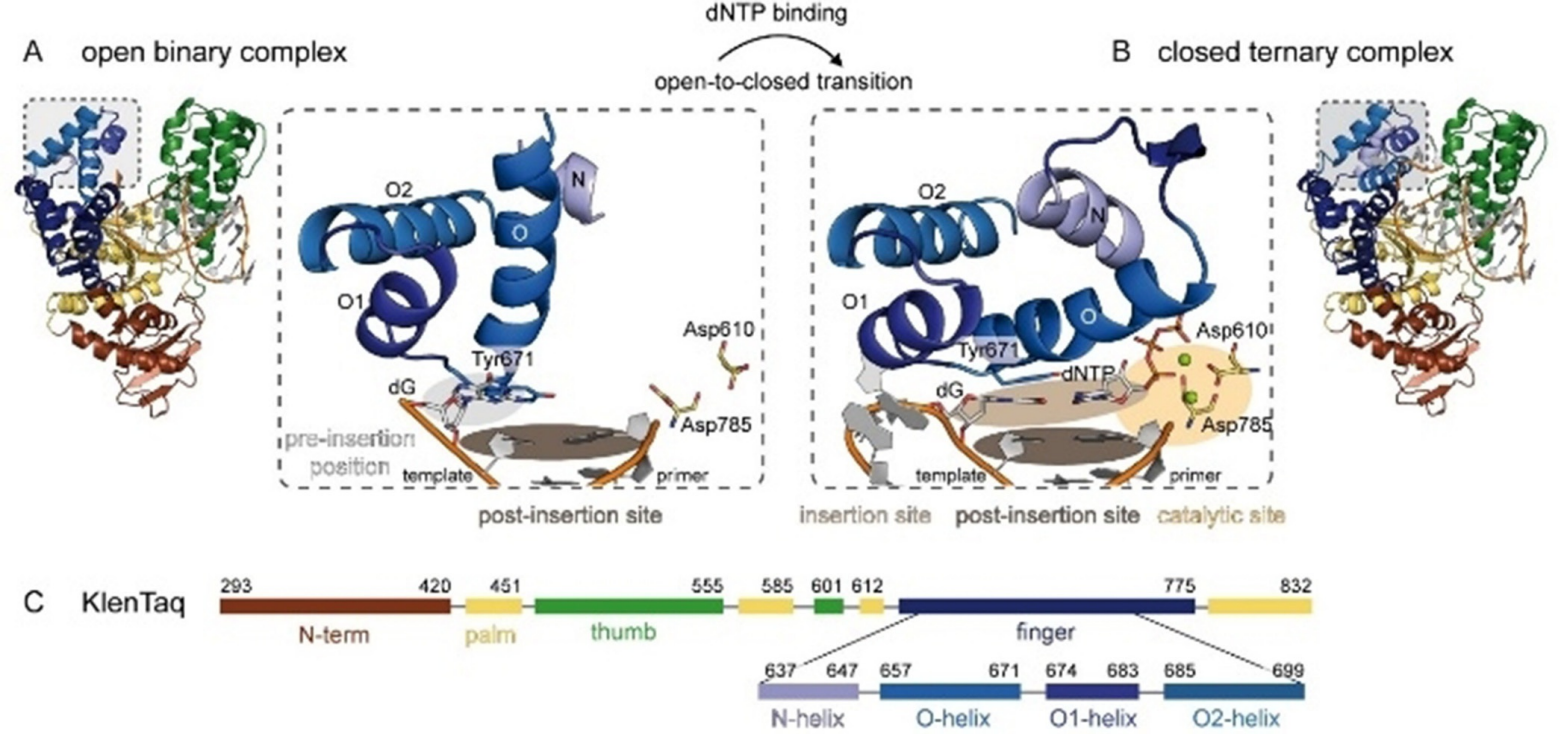
The transition from A) an open binary to B) a closed ternary complex of KlenTaq DNA polymerase upon addition of a triphosphate substrate is visualized. The whole enzyme with N‐terminal, finger, thumb, and palm domains is shown and a close‐up visualizes the part of the finger domain that undergoes the biggest movement (different shades of blue for helices O, O1, O2, and N). The pre‐insertion position, insertion site, post‐insertion site, and catalytic site mentioned in the text are indicated by colored ovals. Mg^2+^ ions are shown as green spheres. C) The subdomain architecture of KlenTaq DNA polymerase and the color code used in A) and B) is given.

KlenTaq DNA polymerase consists of four domains that are termed according to the topology of a hand: the finger, thumb, palm, and N‐terminal domain (Figure 5). Upon DNA binding, the thumb domain closes and together with the finger domain interacts with the primer/template duplex. The first single‐stranded template nucleotide (the templating nucleotide) is situated in an extrahelical, so‐called pre‐insertion position, and a tyrosine residue (Tyr671) stacks on top of the terminal base pair in the post‐insertion site of the duplex DNA. After triphosphate binding, a large conformational change of the finger domain takes place, transferring the enzyme from an open to a closed state (see Figure 5, transition of A to B). During this rearrangement, the tyrosine moves away and the templating nucleotide rotates towards the insertion site where it pairs with the incoming substrate triphosphate. The O‐helix of the finger domain is placed on top of the newly formed base pair. Thereby, a closed complex is formed in which the enzyme can geometrically select (in addition to previous selection steps^68^) for the conserved Watson–Crick structure of the natural base pairs.^65^, ^66^, ^67^, ^71^ The catalytic residues Asp785 and Asp610 are situated in the palm domain and coordinate—together with the triphosphate moiety of the substrate—two magnesium ions (see Figure 5 B). In this arrangement, all components involved in catalysis are positioned in a way that the 3′‐OH group of the primer terminus (which is not present in the crystals) can attack the α‐phosphate of the triphosphate substrate to form a phosphodiester bond. After the reaction, the enzyme translocates on the primer/template strand whereas the newly formed base pair is handed on to the post‐insertion site.

#### 3.4.1 Binary complexes

**Figure 6:**
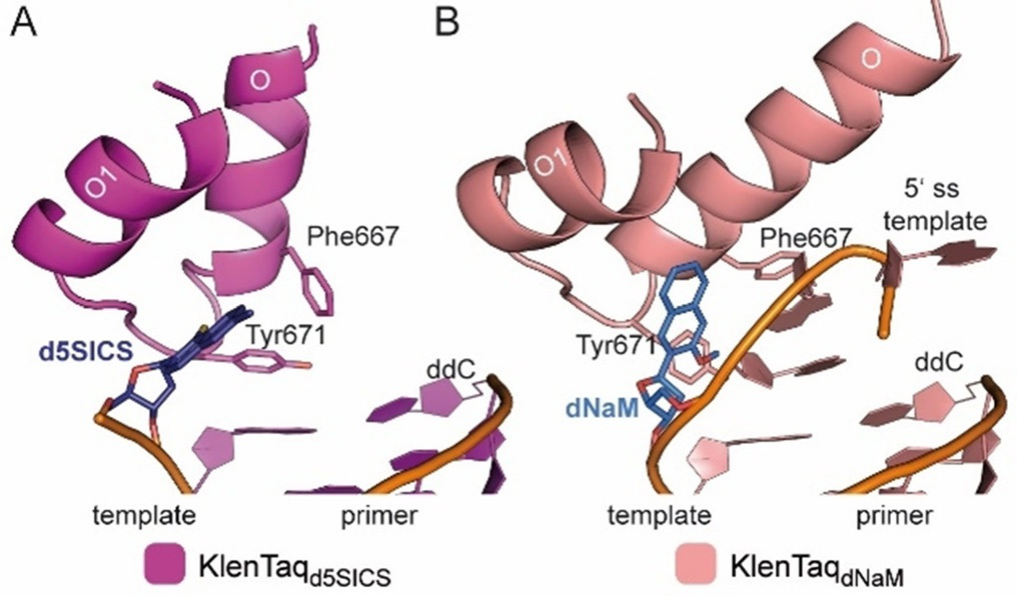
Binary complexes A) KlenTaq_d5SICS_ and B) KlenTaq_dNaM_. The 3′‐primer and 5′‐template nucleotides near the insertion site as well as finger domain helices O and O1 are shown as cartoons. **D5SICS** and **dNaM** are shown as sticks in dark blue and marine, respectively. Tyr671 and Phe667, which are discussed in the text, are shown as sticks (ss=single stranded).

The overall structures of the binary complexes with a templating **dNaM** or a templating **d5SICS** unnatural nucleotide (KlenTaq_dNaM_ and KlenTaq_d5SICS_) are very similar to KlenTaq_dG_ and KlenTaq_dT_. All binary structures are characterized by an open finger domain, which shows flexibility indicated by elevated B‐factors. One significant difference in the structures is the position of the templating nucleotide and the 5′‐single‐stranded template overhang. In KlenTaq_d5SICS_ and KlenTaq_dG_, the templating nucleotides are rotated away from the insertion site and are positioned at the pre‐insertion position pointing towards the solvent (Figure 6 A, shown for KlenTaq_d5SICS_). The three upstream 5′‐single‐stranded template nucleotides are flexible and not resolved in the structures. In KlenTaq_dNaM_ and KlenTaq_dT_, the templating nucleotides are also flipped away from the insertion site, however, to a different position. The single‐stranded template is rotated to the developing DNA duplex where two of the nucleotides stack between the base pair in the post‐insertion site and Phe667 of the finger domain O‐helix (Figure 6 B, shown for KlenTaq_dNaM_). The different arrangements seem to depend on the templating nucleotide and the sequence of the single‐stranded overhang and most probably also its length. Longer single‐stranded overhangs might not undergo the backward rotational movement observed in KlenTaq_dNaM_ and KlenTaq_dT_ and therefore this template arrangement is most probably not relevant in insertion reactions in solution. As the different arrangements are observed for both natural and unnatural templating nucleotides, it is concluded that neither **dNaM** nor **d5SICS** in the templating position perturb the structure of the enzyme in the open state.

#### 3.4.2 Closed ternary complexes

As it was found in NMR studies that **dNaM**–**d5SICS** prefers an intercalated structure in free duplex DNA not resembling a correct natural nucleobase pair, it was difficult to imagine how the efficient replication observed in functional studies can be accomplished by the enzyme. To investigate this circumstance, crystallization of ternary KlenTaq DNA polymerase complexes with hydrophobic UBPs in the active site was desired. Closed ternary complexes were obtained with **dPxTP** paired opposite **dDs** (termed KlenTaq_dDs–dPxTP_) and **d5SICSTP** paired opposite **dNaM** (termed KlenTaq_dNaM–d5SICSTP_) and compared with the fully natural complex KlenTaq_dG–dCTP_. In both complexes, addition of the substrate triphosphate induced the transition from an open to a closed state of the DNA polymerase by closure of the finger domain during which the templating nucleobases are flipped back from their extrahelical positions into the insertion site where the two nucleotides pair. The overall structures are very similar to KlenTaq_dG–dCTP_ with rmsd values for Cα atoms of 0.188 Å and 0.236 Å for KlenTaq_dDs–dPxTP_ and KlenTaq_dNaM–d5SICSTP_, respectively.

**Figure 7:**
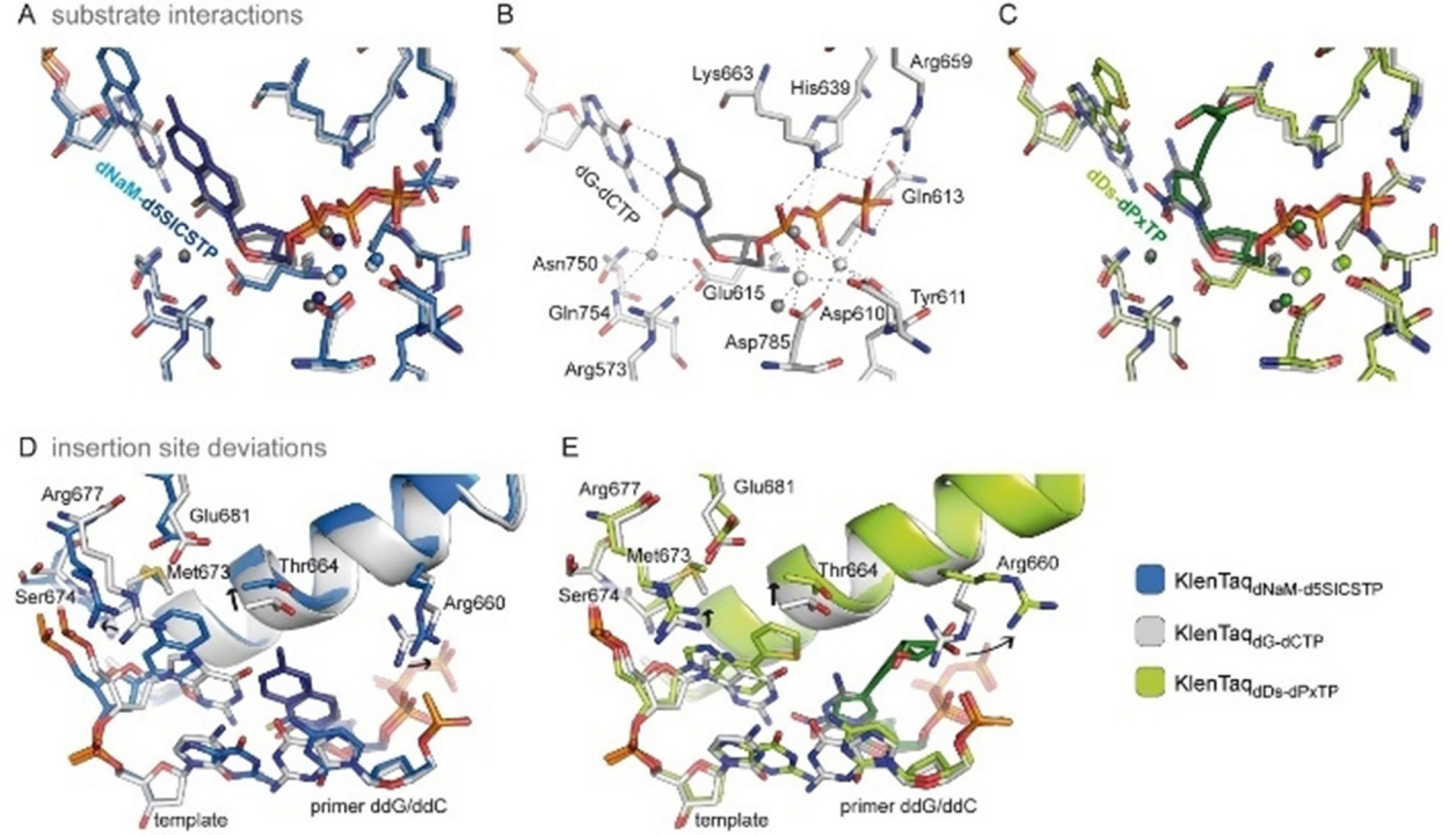
Comparison of ternary complexes KlenTaq_dNaM–d5SICSTP_ (blue), KlenTaq_dDs–dPxTP_ (green) with the fully natural complex KlenTaq_dG–dCTP_ (gray). A–C) Interactions of KlenTaq DNA polymerase with the triphosphate substrate. Interactions are indicated for KlenTaq_dG–dCTP_ by dashed lines in B) and overlays are shown in A) and C). Magnesium ions are shown as spheres and colored marine, light gray, and light green, water molecules are shown as dark‐blue, dark‐gray, and dark‐green spheres, respectively. D,E) Insertion site deviations for KlenTaq_dNaM–d5SICSTP_ and KlenTaq_dDs–dPxTP_ in overlays with KlenTaq_dG–dCTP_. Relevant residues are shown as sticks and the O‐helix is shown as a cartoon. Major movements are indicated by black arrows.

The triphosphate moieties together with Asp610, Asp785, and the backbone of Tyr611 coordinate two magnesium ions, characterizing an active closed complex prior to the insertion reaction (Figure 7 A–C). The distances between the primer 3′‐end (C3′ used for measuring as 3′‐OH is missing) and the α‐phosphate is virtually identical for the two modified complexes and the natural complex (3.8 Å for KlenTaq_dDs–dPxTP_ and KlenTaq_dNaM–d5SICSTP_, and 3.9 Å for KlenTaq_dG–dCTP_). In addition to the metal coordination, the triphosphate substrates seem very well stabilized at their positions through diverse interactions with the enzyme (Figure 7 A–C). The triphosphate moieties interact with the side chains of Lys663, Arg659, and His639 and the backbone of Gln613. On the minor groove side, the nitro group of **dPxTP** and the sulfur atom of **d5SICSTP** engage in water‐mediated interactions with Asn750, Gln754, and Glu615 just as is the case for the O2 atom of **dCTP**. The ribose moiety can form two hydrogen bonds: to the backbone of Glu615 via the 3′‐OH and to Arg573 via the ring oxygen. All these interactions are shown by using dashed lines for KlenTaq_dG–dCTP_ in Figure 7 B. Superimpositions show that these interactions are virtually the same for KlenTaq_dDs–dPxTP_ and KlenTaq_dNaM–d5SICSTP_ (Figure 7 A, C).

**Figure 8:**
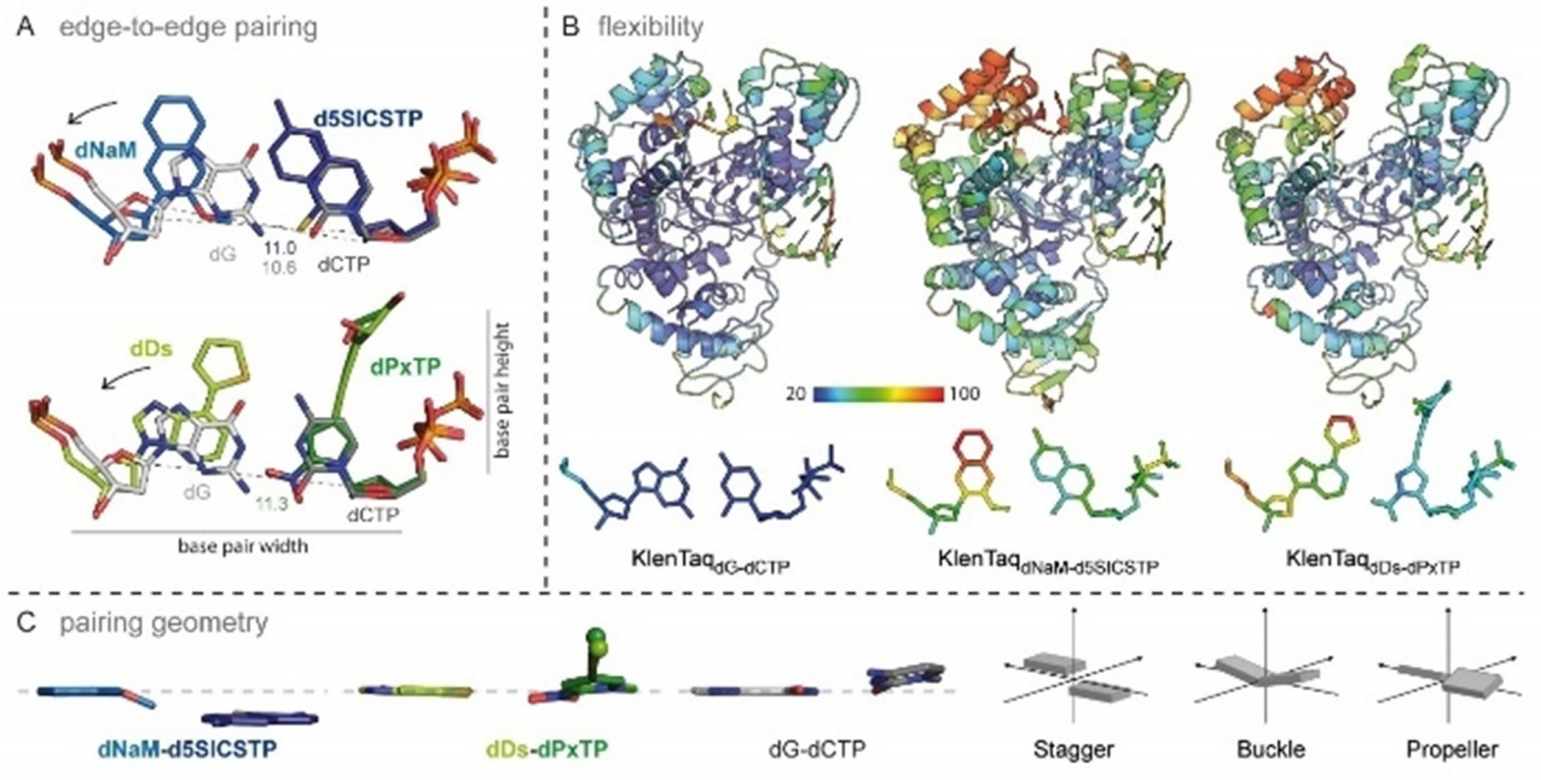
A) Edge‐to‐edge pairing of **dNaM**–**d5SICS** and **dDs**–**dPx** in the active site of KlenTaq DNA polymerase overlaid with the natural **dG**–**dCTP** pair of KlenTaq_dG–dCTP_. The base pair width and height described in the text is indicated. C1′−C1′ distances are given in Å and the enlarged base‐pair widths are indicated by arrows. B) Overall structures and base pair in the insertion site colored by B‐factors (blue=low flexibility, red=high flexibility). C) Base‐pair parameters of hydrophobic UBPs and natural **dG**–**dCTP** are visualized. Ribose and phosphates, as well as diol moiety of **dPx** are omitted for clarity. Base pairs are oriented such that the templating base plane lies orthogonal to the paper plane. Schemes of the parameters, stagger, buckle, and propeller are shown on the right side.

The most interesting finding from our study was that both hydrophobic UBPs do not intercalate but form a coplanar structure similar to the cognate Watson–Crick base pairs (Figure 8 A). The **dNaM**–**d5SICS** pair is positioned edge‐to‐edge (with an average distance of 4.2 Å between the hydrophobic edges of the nucleobases) and a C1′−C1′ internucleotide distance of 11.0 Å. This distance indicates that the pair is slightly enlarged in width compared with the natural **dG**–**dCTP** pair (10.6 Å). The **dDs**–**dPxTP** pair also adopts a planar orientation of the pairing partners. The average distance between the edges is 4.9 Å, resulting in a pair that is even larger in width with a C1′−C1′ distance of 11.3 Å between the pairing partners. To accommodate the wider base pair, in both cases the templating nucleotide is shifted towards the template backbone whereas the triphosphate residues stay in the same well‐defined position found for the natural substrates (Figure 8 A). Along with the shift of the templating nucleotide, interacting amino acids (Arg677, Ser674, and Met673) are also shifted in both structures in a similar way (Figure 7 D, E). To accommodate an artificial base pair with an elevated width, KlenTaq DNA polymerase seems to adjust the insertion site such that only residues on the template side are rearranged but residues in the catalytic site interacting with the triphosphate stay. This behavior would ensure proper alignment of the triphosphate substrate for attack by the 3′‐OH group of the primer end also for pairs that slightly differ in their dimensions from the natural consensus structure. Besides the enlarged base pair width, both hydrophobic pairs have different heights compared with the Watson–Crick pairs (Figure 8 A) although the exact dimensions differ in the two pairs and in their strand context (the orientation of the pairing partners in the primer or template). On the major groove side, the base pair is restricted by the O‐helix of the finger domain. In both structures, the larger base moieties cause a small shift of the overall O‐helix and connected helices away from the insertion site whereas the enzyme residues confining the base pair on the minor groove side stay unperturbed (Figure 7 D, E). More specifically, Thr664, which is situated closest to the thiophenyl moiety of **dDs** and to both **dNaM** and **d5SICSTP**, shifts upwards by 0.6 Å (measured at Cα atoms) in KlenTaq_dDs–dPxTP_ and 0.9 Å in KlenTaq_dNaM–d5SICSTP_. To accommodate the propynyldiol moiety of **dPxTP**, Arg660, located at the N‐terminus of the O‐helix, shifts away in a similar way as already observed for other KlenTaq structures with modified substrates (Figure 7 E).^72^, ^73^, ^74^ Owing to the shift, an interaction of Arg660 with the 3′‐primer end is lost. This interaction could be important to stabilize the closed enzyme by connecting the finger domain and the bound DNA duplex. The lost interaction could theoretically be replaced by the diol moiety of **dPxTP**. Along with the observed shift, the tip of the finger domain as well as the artificial base pairs in KlenTaq_dDs–dPxTP_ and KlenTaq_dNaM–d5SICSTP_ show higher flexibility compared with the rest of the enzyme, indicated by elevated B‐factors (Figure 8 B). Apparently, the finger domain cannot close as tightly as in the natural complex, which could explain why the studied hydrophobic artificial base pairs are still formed with somewhat diminished efficiency compared with the natural counterparts.

The more detailed orientation of the hydrophobic pairing partners with respect to each other can be described by so‐called base pair parameters. These parameters were determined by using the 3DNA webserver.^75^ As the artificial nucleotides are not recognized by the software, **dG** and **dCTP** were superposed manually in the program Coot^76^ on the artificial nucleotides fitting the glycosidic bond and the nucleobase plane. The propeller twist (relative torsion between pairing partners with respect to the base pairing axis) and the buckle angle (angle of the bend between the two base planes across the line of base pairing) is similar between **dDs**–**dPxTP** and **dG**–**dCTP** but not to **dNaM**–**d5SICSTP** where it is significantly smaller (Figure 8 C). In addition, the **dNaM**–**d5SICS** pair exhibits a larger relative shift of the bases along the *z* axis (stagger=−1.2 Å) compared with **dG**–**dCTP** and **dDs**–**dPxTP**, where the stagger is close to zero. We show that although both artificial base pairs studied adopt an edge‐to‐edge orientation just as natural pairs in the active site of KlenTaq DNA polymerase, the base pair non‐planarity parameters can still vary between the different base pair candidates. All‐in‐all, concerning the three base pair non‐planarity parameters, buckle, propeller, and stagger, the **dDs**–**dPxTP** pair is more similar to the natural **dG**–**dCTP** pair in the active site of KlenTaq than **dNaM**–**d5SICSTP**.

In summary, binding of **d5SICSTP** opposite **dNaM** as well as **dPxTP** opposite **dDs** to KlenTaq DNA polymerase induces the formation of a closed enzyme complex that is poised for catalysis. In this complex, the sum of interactions between the developing artificial base pairs and the active site of KlenTaq DNA polymerase seem to well stabilize the hydrophobic pairs in the natural Watson–Crick‐like geometry. This finding thereby explains the high incorporation efficiencies of hydrophobic artificial base pairs by DNA polymerases despite lacking connecting hydrogen bonds.

#### 3.4.3 Partially closed ternary complexes

**Figure 9:**
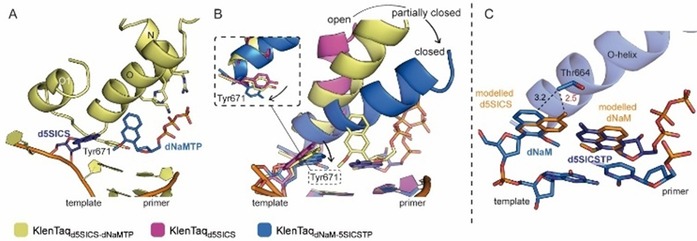
A) Partially closed ternary complex KlenTaq_d5SICS–dNaMTP_ (pale yellow). Residues of the O‐ and N‐helix interacting with the triphosphate are shown as sticks and **d5SICS** and **dNaMTP** are shown in dark blue and marine, respectively. B) Position of the O‐helix, the templating nucleotide, and Y671 (inset in a different orientation to visualize differences) for the open complex KlenTaq_d5SICS_ (pink), the partially closed complex KlenTaq_d5SICS–dNaMTP_ (pale yellow), and the fully closed complex KlenTaq_dNaM–d5SICSTP_ (blue) are shown. Transitions are indicated by black arrows. The substrates **dNaMTP** (for the partially closed complex) and **d5SICSTP** (for the closed complex) are shown in pale yellow and dark blue, respectively. C) Model of a **d5SICS**–**dNaMTP** pair (orange) superposed on the **dNaM**–**d5SICSTP** pair in the closed KlenTaq_dNaM–d5SICSTP_ structure (blue). A potential clash with Thr664 is indicated in red. Distances are given in Å.

In contrast to the fully closed complexes KlenTaq_dNaM–d5SICSTP_ and KlenTaq_dDs–dPxTP_, a different reaction state was trapped in the complex KlenTaq_d5SICS–dNaMTP_ with the opposite sequence context compared with KlenTaq_dNaM–d5SICSTP_ (now **d5SICS** in the template and **dNaMTP** added as substrate).^69^ Soaking of binary crystals containing **d5SICS** at the templating position with **dNaMTP** led to a ternary complex, however, the transition to a closed ternary complex did not fully take place. The finger domain is trapped in a partially closed conformation and the **dNaMTP** substrate is bound to the O‐helix where it appears to be stabilized by ionic interactions of the triphosphate moiety with different protein residues (Figure 9 A). Consistent with the finger domain being positioned in an intermediate conformation between the fully open and fully closed states, Tyr671 is slightly displaced from its open conformation position in the insertion site (inset in Figure 9 B) and the templating **d5SICS** slightly moves from its extrahelical position towards the insertion site. Similar partially closed complexes have been described for BF DNA polymerase (Klenow‐like fragment of *Bacillus stearothermophilus* DNA polymerase I) with mismatched nucleotides in the insertion site.^68^ It has been suggested that this conformation is a pre‐selection state in which the DNA polymerase tests for complementarity between the incoming substrate and the templating nucleotide before transitioning to the closed catalytically competent state. Additionally, partially closed ternary structures are reported for KlenTaq with an abasic site analog in the templating position.^77^, ^78^ Apart from these structural observations, a partially closed state was also found in Förster resonance energy transfer (FRET) studies for the homologous *E. coli* DNA polymerase I.^79^, ^80^, ^81^, ^82^ It has been shown that the intermediate state is especially favored in the case of an incorrect nucleotide or ribonucleotide substrates bound to the enzyme and only sparsely populated with complementary dNTPs. Therefore, it is suggested that the state is a primary checkpoint for nucleotide selection on the pathway to the chemical step.^83^ For efficient **dNaMMP** incorporation, we assume that a similar planar arrangement as observed in KlenTaq_dNaM–d5SICSTP_ is adopted, which would be reached by additional conformational changes based on our partially closed structure. The fact that for **dNaM**–**d5SICSTP**, a fully closed complex was readily obtained but for the **d5SICS**–**dNaMTP** only the described intermediate complex was trapped, is consistent with the often lower insertion efficiency of **dNaMMP** opposite **d5SICS** compared with **d5SICSMP** opposite **dNaM**.^84^ The lower incorporation efficiency could be explained by changes or clashes in the active site of a fully closed enzyme with the **d5SICS**–**dNaMTP** pair. A superimposition of the **d5SICS**–**dNaMTP** pair on the **dNaM**–**d5SICSTP** pair in the closed KlenTaq_dNaM–d5SICSTP_ reveals that the methyl group of the **d5SICS** nucleobase in the templating position could come close to the O‐helix residue Thr664 (Figure 9 C).^85^ This potential clash might make it more difficult for the finger domain to fully close. The base moiety, however, should still have enough freedom to rotate around the glycosidic bond, which would enable a closed complex but with additional energetic penalty.

#### 3.4.4 Post‐chemistry extension complexes

**Figure 10:**
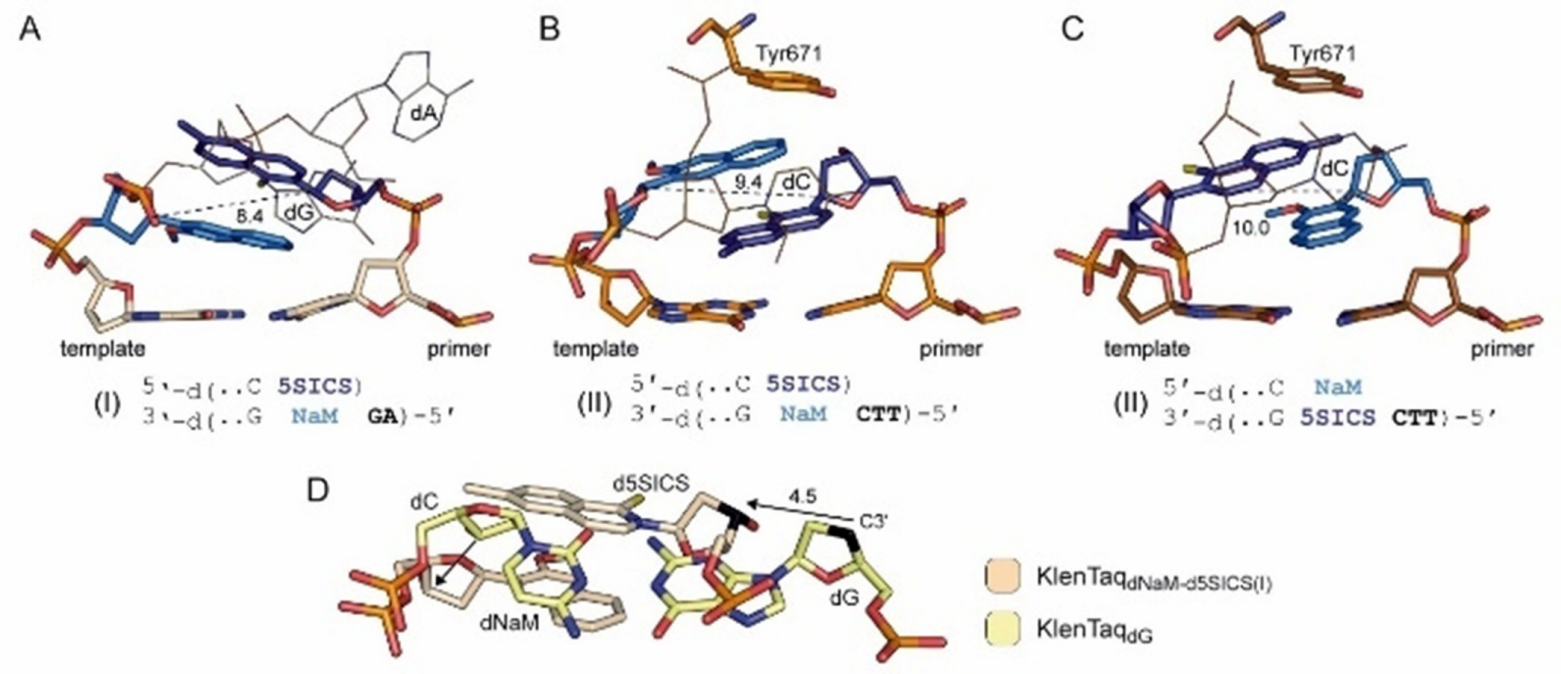
A–C) Intercalating structures of **dNaM**–**d5SICS** at the primer/template end in two different sequence contexts (I and II). For sequence context II, the structure was solved in both strand contexts (**dNaM** in the primer and **d5SICS** in the template and vice versa; B and C). C1′−C1′ distances between **dNaM** and **d5SICS** (marine and dark blue, respectively) are given in Å. 5′‐Single‐stranded template overhanging nucleotides are shown as lines and are indicated in bold letters in the sequence below the structures. D) Displacement of the intercalating primer and template nucleotides is exemplarily shown for KlenTaq_dNaM–d5SICS(I)_ (sand) compared with the natural situation in KlenTaq_dG_ (pale yellow). C3′ atoms of the primer terminus are shown in black and the displacements in the primer and template are indicated by black arrows.

To understand the process of elongation after an UBP is formed in a DNA duplex, binary crystal structures of KlenTaq with **dNaM**–**d5SICS** in the post‐insertion site in both strand contexts (**dNaM** in the template and **d5SICS** in the primer and vice versa) and in different sequence contexts were solved (Figure 10 A–C).^69^ The structures reveal that after synthesis and transition of the closed to the open enzyme complex, the artificial pair forms an intercalated structure similar to the one observed in the NMR study of free duplex DNA containing **dNaM**–**d5SICS** (see section 3.3).^55^ With either a **dG** or **dC** nucleotide 5′ to the templating nucleotide mainly two different modes of intercalation are observed (Figure 10 A–C, different sequence contexts are termed I and II here). With a dG 5′ to the template dNaM, the d5SICS at the primer terminus is placed on top of dNaM in the template (Figure 10 A). With a dC 5′ to dNaM or d5SICS in the template, the hydrophobic primer nucleotide is placed below its pairing partner (Figure 10 B, C). All intercalating structures are characterized by a decreased C1′−C1′ distance of the pairing partners (between 8.4 and 10.0 Å). Both intercalation modes show unique stabilization patterns with surrounding protein residues (for more details, see ref. 69). As a consequence of intercalation in the post‐insertion site, shifts in the thumb domain and the position of the primer/template duplex are observed. These become apparent in an overlay of the post‐insertion complexes with the natural binary complexes, exemplarily shown for the KlenTaq complex with **dNaM**–**d5SICS** and the template 5′ overhang with the sequence “GA” (termed KlenTaq_dNaM–d5SICS(I)_) and KlenTaq_dG_ (Figure 10 D). The primer as well as the template nucleotides are shifted compared with their natural position. The 3′‐OH group responsible for the next insertion reaction shifts by 4.5 Å (arrow in Figure 10 D) and to a similar extent in the other post‐insertion complexes. Based on the observed arrangement, extension of the primer with the next incoming nucleotide seems difficult. It is assumed that reversal of the intercalation of the artificial pairing partners has to take place before or during finger domain closure after binding of a new cognate substrate. The necessity of large conformational changes before extension of the hydrophobic artificial base pairs likely explains the low extension efficiencies observed in primer extension reactions, rendering the extension the bottleneck in the replication of DNA containing the UBP.^86^

#### 3.4.5 Proposed mechanism of replication for hydrophobic UBPs and its consequences

**Figure 11:**
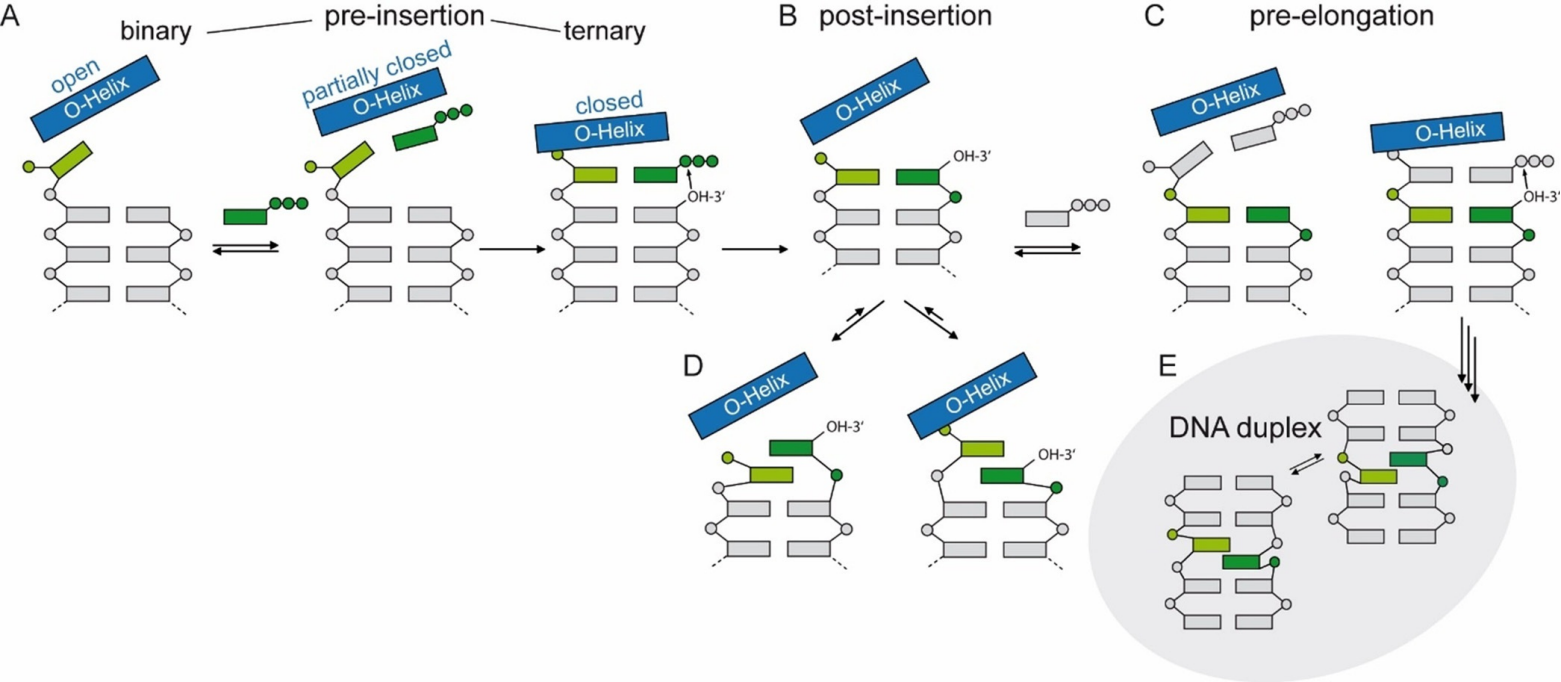
Scheme of the proposed mechanism of replication for hydrophobic artificial base pairs. The steps corresponding to incorporation of the unnatural monophosphate (dark green) and subsequent extension of the nascent unnatural base pair are shown. Thereby, the O‐helix of the protein is shown as blue rectangles, phosphates are indicated with circles, natural nucleosides are shown as gray rectangles, and unnatural nucleotides are shown as dark‐ and light‐green rectangles. The structure of the unnatural pair in free duplex DNA^53^, ^54^ after several rounds of extension and enzyme dissociation is shown in the gray oval. Figure adapted from reference 83.

Based on the described data of the pre‐insertion and the elongation complexes of the **dNaM**–**d5SCIS** pair as well as previously reported kinetic and structural data,^65^, ^66^, ^67^, ^68^ a replication mechanism for hydrophobic artificial base pairs was proposed (Figure 11). In a first step, the hydrophobic substrate binds to the O‐helix after which the enzyme samples different conformations and transitions to a closed state as soon as sufficiently stabilizing hydrophobic and packing interactions are made (Figure 11 A). The enzyme closure in turn induces the UBP to adopt a planar, Watson–Crick‐like structure that fits into the constraints of the active site and enables insertion of the substrate into the growing primer strand. Depending on the substrate bound, the intermediate states can be more or less transient. In the case of a bound **dNaMTP**, the crystallographically trapped partially closed state somehow seems to be more stable than the corresponding closed complex. After the insertion reaction, the DNA polymerase returns to the open state and pyrophosphate is released.^87^ In this state, the UBP adopts a cross‐strand intercalated structure, which would hamper continued primer elongation (Figure 11 B, D). It is assumed that additional thermal fluctuations are necessary to resolve intercalation of the terminal base pair and reorganization of the DNA polymerase active site before the next nucleotide can be incorporated (Figure 11 C). The postulated mechanism clearly shows that the critical reaction step is the elongation step as reversal of intercalation and reorganization of the active site is needed to continue the synthesis (Figure 11 C). To overcome this drawback, it was concluded that intercalation properties have to be reduced to ease elongation with natural nucleotides. This can be realized, for example, by reducing the aromatic surface area of the nucleobases. In addition, interstrand intercalation should also be reduced by favoring intrastrand packing (meaning stabilization of unnatural pairing partners with natural nucleotides in the same strand) as opposed to interstrand packing.^42^ Both concepts were realized by the Romesberg group in the development of **dTPT3** as a pairing partner for **dNaM** (Figure 1 B). Distal ring contraction and heteroatom derivatization of **d5ISCS** resulted in the **dTPT3** nucleotide, which shows improved incorporation and elongation properties.^12^ Whether the **dMaM**–**dTPT3** adopts an intercalated post‐insertion structure and to what extent is not known. If intercalation occurs, it is possible that the arrangement is resolved more easily owing to its lower stability. This might be the reason for the higher PCR efficiency and fidelity observed for **dNaM**–**dTPT3** compared with **dNaM**–**d5SICS**. Similar variations as for **d5SICS** to yield **dTPT3** were made for **dNaM** and yielded the nucleotides **dMTMO** and **dPTMO** carrying a thiophene moiety (Figure 2). As mentioned above, the base pairs **dMTMO**–**dTPT3** and especially **dPTMO**–**dTPT3** show improved in vivo retention rates compared with **dNaM**–**dTPT3** despite their inferior properties in vitro.^42^ A modeling study suggests that interaction of the thiophene sulfur atoms of both **dMTMO** and **dPTMO** favors internucleotide interactions with the primer nucleotide, which in turn disfavors intercalation.^42^ For **dCNMO**, which bears a smaller, single ring nucleobase (Figure 2), pairing with **dTPT3** also shows superior in vivo performance compared with **dNaM**–**dTPT3**. It is assumed that the smaller ring is less prone to cross‐stand intercalation and the pair more likely adopts an edge‐to‐edge structure even without the constraints of the closed DNA polymerase.^43^

## 4 Structure of Hydrogen‐Bonding UBPs: The Benner Pair(s)

Besides the discussed hydrophobic base pairs, the second well‐replicated base pair family consists of the pairs developed in the Benner lab. The structure of the best candidate, the **dP**–**dZ** pair, in free duplex DNA and in the active site of KlenTaq DNA polymerase was also studied.^32^, ^88^, ^89^, ^90^

### 4.1 Structure in free duplex DNA

**Figure 12:**
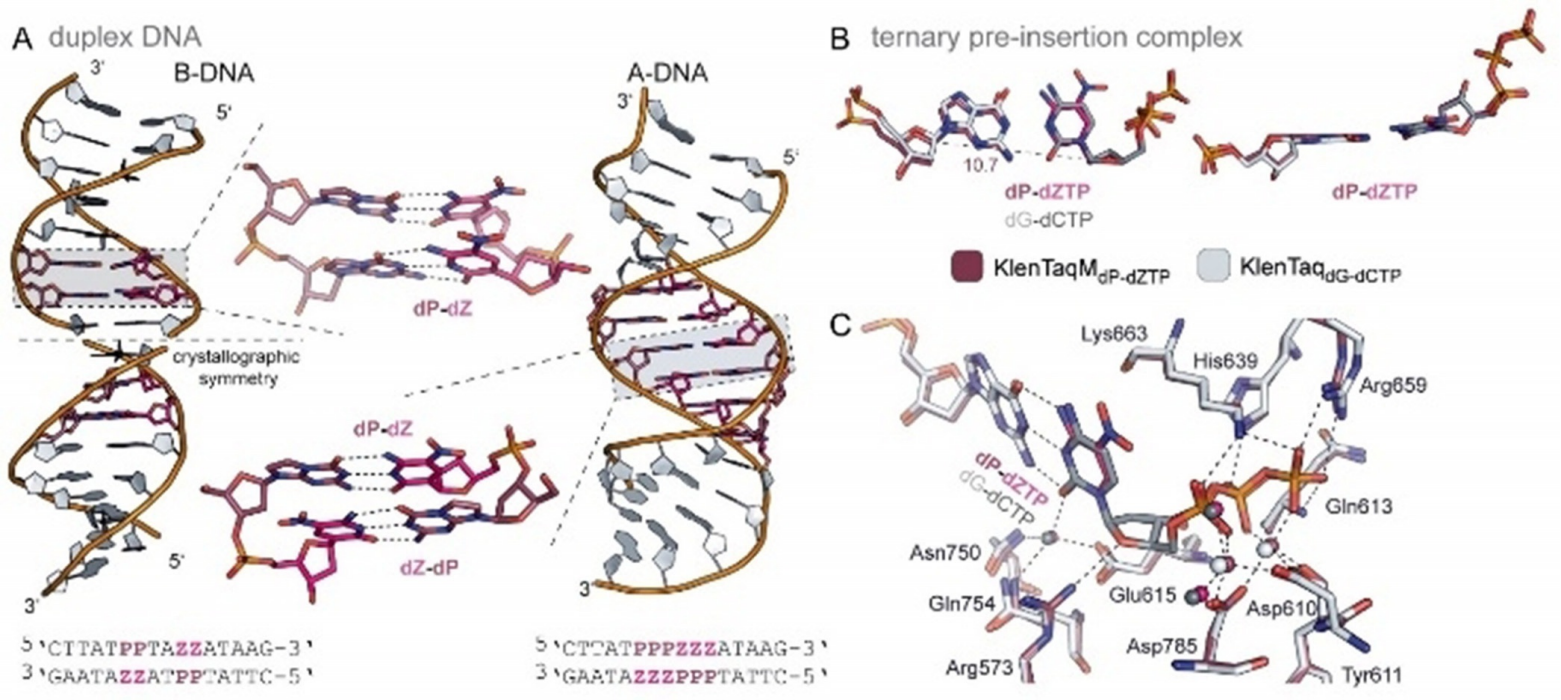
A) Structure of **dP**–**dZ** in free duplex DNA for two different sequences. The duplexes forming B‐DNA or A‐DNA are shown as cartoons and the artificial nucleotides **dP** and **dZ** are colored raspberry and pink, respectively, and are shown as sticks in a close‐up representation. Sequences used for crystallization are shown below the duplexes. B,C) Superimposition of KlenTaqM_dP–dZTP_ (raspberry) and KlenTaq_dG–dCTP_ (gray). B) Structure of the **dP**–**dZTP** pair and the natural **dG**–**dCTP** pair in two different orientations. C1′−C1′ distance is given in Å. C) Residues interacting with the substrate triphosphate as well as coordinating magnesium ions (raspberry and light gray) and water molecules (pink and dark gray) are shown. Interactions are indicated by dashed lines.

The **dP**–**dZ** pair does not significantly perturb the double helical structure of DNA. Crystallographic studies showed that within a 16‐mer DNA double‐strand **dP** hydrogen bonds with **dZ** with geometries and distances similar to the canonical base pairs and the DNA duplex adopts known helical forms: A‐form for six consecutive **dP**–**dZ** pairs and mostly B‐form for two consecutive **dP**–**dZ** pairs (Figure 12 A).^88^ One characteristic is that the major groove width is enlarged by up to 1 Å with respect to comparable G–C pairs in A‐ and B‐DNA, which may be necessary to accommodate the nitro group on **dZ**. Another unique feature is the stacking interaction of the nitro group in **dZ** with the adjacent nucleobase in the A‐form duplex. Even with the addition of a different UBP of the AEGIS family (the **dB**–**dS** pair, Figure 1 C), the double helix structure remains intact.^90^ The three studied 16‐mer DNA duplexes consisting of four different base pairs (including six consecutive UBPs) only show minor geometrical differences compared with unmodified DNA.^90^ This sequence‐independent structural regularity is attributed to a big extent to the presence of hydrogen bonding in the UBPs and is a key prerequisite for different molecular biological applications.

In addition to these crystallographic studies, the DNA duplex containing six consecutive **dP**–**dZ** pairs was studied by using long timescale (50 μs) molecular dynamics (MD) simulations.^89^ Here, a significantly wider major groove and differing average values of stagger, as well as the dinucleotide step parameters, slide, twist, and h‐twist, as observed for an analogous natural oligonucleotide were identified (for more details, see ref. 89). Interestingly, a cumulative effect of the number of **dP**–**dZ** pairs on the major groove width was observed. This finding could imply that inclusion of a large number of consecutive **dP**–**dZ** nucleobase pairs could result in an unstable DNA double helix.

### 4.2 Structure in KlenTaq DNA polymerase

The acceptance of the **dP**–**dZ** pair by DNA polymerases was studied by using X‐ray crystallography.^32^ Therefore, a KlenTaq mutant (M444V, P527A, D551E, and E832V) that showed improved incorporation of **dZMP** opposite **dP** was used.^31^ More specifically, a closed ternary pre‐insertion complex with **dZTP** paired opposite templating **dP** (KlenTaqM_dP–dZTP_) and a post‐incorporation complex with **dP**–**dZ** at the primer/template end in an open binary complex (KlenTaqM_dP–dZ_) was trapped (for primer/template sequence and PDB codes, see Figure 5).

The overall structure of KlenTaqM_dP–dZTP_ in the insertion site is similar to KlenTaq_dG–dCTP_ (rmsd: 0.347 for Cα atoms). As expected, **dP** and **dZTP** pair through three hydrogen bonds and the pair is oriented edge‐to‐edge with similar geometric parameters as **dG**–**dCTP** (Figure 12 B). The base pair width characterized by the C1′−C1′ distance is virtually identical between **dP**–**dZTP** (10.7 Å) and **dG**–**dCTP** (10.6 Å). Similar interactions for the enzyme, the primer/template duplex, and the incoming dNTP as in KlenTaq wild‐type (WT) with natural substrates are found (Figure 12 C). As a main difference of KlenTaqM_dP–dZTP_ compared with KlenTaq_dG–dCTP_, the Benner group identified a larger closure angle of the mutant′s finger domain when comparing the transitions of the mutant and WT binary to ternary complexes. Higher B‐factors at the tip of the finger domain, however, compared with KlenTaq_dG–dCTP_, and the fact that parts of the finger domain could not be modeled, indicate a less stable closed complex, which is similar to our finding in KlenTaq_dDs–dPxTP_ and KlenTaq_dNaM–d5SICSTP_.

**Figure 13:**
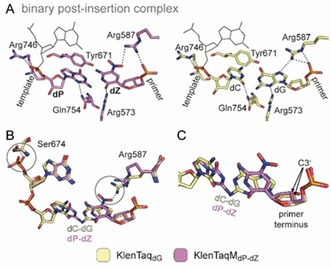
A) Minor and major groove interactions of KlenTaq with the terminal primer/template pair in the binary complexes KlenTaq_dG_ (pale yellow) and KlenTaqM_dP–dZ_ (violet). B,C) Superimposition of KlenTaq_dG_ and KlenTaqM_dP–dZ_ to visualize potential clashes if **dP**–**dZ** is formed in the WT enzyme (B) and to visualize differences in the position of the primer terminus (C, indicated by arrows).

The overall structure of the binary post‐insertion complex KlenTaqM_dP–dZ_ is again similar to the binary complex KlenTaq_dG_ (rmsd: 0.334 for Cα atoms). Minor groove and major groove interactions of the respective terminal base pair with the enzyme are almost identical for either the UBP or a natural base pair (Figure 13 A). In a superimposition of the two structures (for details, see ref. 32), the Benner group identified relevant differences in the template region in the vicinity of the active site (Figure 13 B, circled regions). The slightly different positioning of the phosphate moiety of the templating dG and presence of the nitro group of **dZ** would cause two clashes within the WT structure. Therefore, it is concluded that the post‐incorporation product (**dP**–**dZ** in the post‐insertion site) presents a challenge for the WT enzyme, which would need to be resolved by additional movements within the enzyme. The position of the ribose C3′ carrying the catalytic 3′‐OH at the primer terminus, however, is only slightly displaced when comparing KlenTaqM_dP–dZ_ and KlenTaq_dG_ (Figure 13 C). This is in great contrast to the binary post‐insertion complexes with the hydrophobic **dNaM**–**d5SICS** pair (Figure 10 D).

## 5 Comparison of Different UBP Candidates

Apart from their molecular structure and pairing concept, the three UBP candidates discussed in this review differ in their processing by DNA polymerases, their structure in the active site of KlenTaq DNA polymerase, and their structural influence on free duplex DNA. Thereby, pronounced differences exist between the two families: hydrogen‐bonding versus non‐hydrogen‐bonding base pairs, but some differences are also present within the group of hydrophobic UBPs.

### 5.1 Comparison of hydrophobic UBPs

#### 5.1.1 Processing by DNA polymerases

The Hirao pairs **dDs**–**dPx** (meaning **dDs** and differently modified **dPx**) are replicated more efficiently by a B‐family DNA polymerase (Deep Vent *exo*+) whereas for the Romesberg pairs (**dNaM**–**d5SICS** and related pairs), the best in vitro results are obtained with a mixture of Taq (family A) and Deep Vent DNA polymerase (family B).^36^, ^37^, ^40^ As replicative DNA polymerases share a common selection mechanism,^66^ our structural studies—although made with the A‐family KlenTaq DNA polymerase—can explain the high incorporation efficiency and selectivity that is reached by other DNA polymerases. We act on the assumption that the utilized B‐family DNA polymerase Deep Vent (and of course also the full length Taq DNA polymerase) also enforces a Watson–Crick‐like pairing of the hydrophobic artificial base pairs upon closure of the finger domain. The different UBP acceptance by different members of A‐ and/or B‐family polymerases suggests that this parameter should be considered in optimizing in vitro amplification conditions for new candidates.

#### 5.1.2 Structure in the active site of KlenTaq

Regarding the ternary structures KlenTaq_dDs–dPxTP_ and KlenTaq_dNaM–d5SICSTP,_ only small differences are observed within the two UBP/enzyme complexes, but we find similar differences compared to the fully natural complex. Both hydrophobic artificial base pairs have an elevated base pair width and height and amino acid side chains shift on the template and the major groove side of the UBPs in both cases. The finger domain is more flexible in both UBP/enzyme complexes compared with the natural complex, which indicates that the unnatural pair leads to a less stable closed complex, explaining the still lower insertion efficiency of these unnatural substrates. In a detailed analysis regarding the base pair parameters,^70^ the Hirao pair **dDs*–*dPxTP** in the active site of KlenTaq_dDs–dPxTP_ is more similar to the natural **dG**–**dCTP** pair than **dNaM**–**d5SICSTP** in KlenTaq_dNaM–d5SICSTP_, which, however, does not lead to significantly better incorporation properties.

### 5.2 Comparison of hydrogen‐bonding and hydrophobic UBPs

#### 5.2.1 Processing by DNA polymerases

Generally, the hydrogen‐bonding **dP**–**dZ** pair shows less amplification fidelity in PCR experiments compared with the hydrophobic UBPs discussed here (see values in section 2.1), mainly owing to mispairing with natural nucleotides.^30^, ^33^ Therefore, in terms of orthogonality to natural pairs, it seems to be advantageous if pairing relies on a different principle. The reported pairs that rely on hydrophobic and packing interactions rather than hydrogen bonds show low incorporation efficiencies opposite natural pairs, leading to high fidelities in replication. If, however, several consecutive UBPs should be inserted in a DNA strand, hydrogen‐bonding UBPs perform explicitly better. Although enzymatic incorporation of up to four consecutive **dP**–**dZ** pairs into a DNA strand can be accomplished,^30^ consecutive incorporation of hydrophobic UBPs is more difficult. In case of **dDs**–**dPx**, highly efficient amplification could only be reached if two **dDs** bases were separated by at least six natural bases.^38^ For the **dNaM**–**d5SICS** pair, sequences containing two consecutive unnatural pairs or two unnatural pairs separated by one or six natural nucleotides could indeed be amplified but with lower fidelity than with only one UBP in the investigated DNA strand.^40^ This different fidelity most probably relies on the two different types of pairing: hydrogen‐bonding opposed to hydrophobic and stacking interactions, however, hydrogen‐bonding is advantageous in this case.

#### 5.2.2 Structure in duplex DNA

Pairings based on hydrophobic and packing interactions favor intercalation of the pairing partners under some conditions, for example, in free duplex DNA in the reported NMR studies^55^, ^56^ or in post‐incorporation complexes with KlenTaq DNA polymerase,^69^ which distorts the structure of the DNA double helix at the site of the UBP. In contrast, even several consecutive **dP**–**dZ** pairs do not destroy the helical structure of a DNA duplex.^88^ Albeit, also here the three‐dimensional structure is affected (wider major groove and differing step and helix parameters than observed for the analogous natural oligonucleotide), as was shown by molecular dynamics simulations^89^ and crystal structures.^88^ Experimental structures of free duplex DNA containing two or more consecutive hydrophobic artificial base pairs do not exist to our knowledge. However, MD simulations by Galindo‐Murillo et al. show that the double helical structure of DNA is disturbed to a great extent if more than one **dNaM**–**d5SICS** pair is included in the DNA and completely collapses into a globular structure with five UBPs present in the sequence.^54^

#### 5.2.3 Structure in the active site of KlenTaq

In the closed ternary complexes, the three UBPs **dP**–**dZ**, **dNaM**–**d5SICS**, and **dDs**–**dPx** behave similarly. All pairs adopt a Watson–Crick‐like planar edge‐to‐edge structure and induce the DNA polymerase to close. The enzyme establishes interactions with the triphosphate substrate through the same residues in the three complexes. In contrast to the hydrophobic UBPs, **dP**–**dZ** does not show an elevated base pair width and only a difference in height owing to the nitro group of the substrate **dZ**, which does not seem to disturb the closure of the finger domain. Base pair geometric parameters (stagger, buckle, and propeller) are similar for **dP**–**dZTP**, **dDs**–**dPxTP**, and **dG**–**dCTP** but differ in the case of **dNaM**–**d5SICSTP**. This difference, however, does not seem to directly influence the incorporation efficiency as **dNaM**–**d5SICSTP** is well replicated by the related Taq DNA polymerase.^12^

A significant difference exists regarding the binary post‐insertion complexes with either the hydrophobic **dNaM**–**d5SICS** pair or the hydrogen‐bonding **dP**–**dZ** pair. Intercalation of **dNaM** and **d5SICS** in the post‐insertion site distinctly distorts the primer/template duplex and we assume that large conformational rearrangements are necessary to enable an elongation reaction. In KlenTaqM_dP–dZ_, the primer 3′‐end is not significantly different compared with the natural complex and elongation seems easier.

## 6 Summary and Conclusions

In this review, we discussed two different families of artificial base pairs. The **dP**–**dZ** pair is based on an alternative hydrogen‐bonding pattern (rearranged hydrogen‐bonding donor and acceptor groups) compared with the natural base pairs **dA**–**dT** and **dG**–**dC** and adopts similar structures in free duplex DNA and the active site of KlenTaq DNA polymerase as the natural pairs. Therefore, known DNA double helical forms (A‐ and B‐DNA) are adopted even if several consecutive **dP**–**dZ** or related pairs are present in a DNA strand. Compared with completely natural DNA, however, several structural parameters that characterize the double helix differ, resulting, for example, in a double helix with a wider major groove. The fidelity of replication by DNA polymerases is still lower for **dP**–**dZ** than for the discussed hydrophobic UBPs, mainly owing to the higher propensity for mispairing with natural nucleotides.

The second family, the hydrophobic UBPs, are different in structure and pairing mechanism compared with the natural nucleotides. Nevertheless, both **dNaM**–**d5SICS** and **dDs**–**dPx** are replicated with high fidelity in PCR reactions. Our structural studies of KlenTaq and **dNaM**–**d5SICS** and **dDs**–**dPx** emphasize that the pairs relying on hydrophobic and packing forces are sufficiently plastic to adopt the edge‐to‐edge structure necessary for positive selection by a DNA polymerase in the insertion site. In free duplex DNA or at the post‐insertion site within the binary DNA polymerase/primer/template complex, in contrast, **dNaM**–**d5SICS** pairs in an intercalative mode where stacking interactions between the pairing partners seem to be maximized.^55^, ^69^ This is in contrast to the later performed computational studies, which showed that in free duplex DNA, **dNaM**–**d5SICS** and related pairs adopt a rather planar orientation. For **dDs**–**dPx**, structures in free duplex DNA or post‐insertion KlenTaq complexes do not exist. Partly inspired by structural data and the proposed mechanism of replication for hydrophobic artificial base pairs, the optimized **dNaM**–**dTPT3** pair was developed in the Romesberg group, which is PCR amplified with even higher fidelities compared with **dNaM**–**d5SICS**. Insertion of consecutive hydrophobic UBPs or several hydrophobic UPS separated by only a few natural nucleotides into a DNA strand is still challenging. This is consistent with MD simulations, which show that DNA strands containing several hydrophobic UBPs do not form stable DNA double helices. For many applications (e.g., the coding for unnatural amino acids), however, the presence of several UBPs within a short sequence is not necessary. An additional feature of the **dDs**–**dPx** pair is that DNA containing the dPx nucleotide can be further modified with functional groups of interest through Schiff base formation involving the diol moiety.

All described artificial base pairs and potential emerging ones show different properties and are useful in diverse applications. Each of these pairs has got its own advantages or disadvantages, which definitely support their parallel existence. Fields of application are manifold. This motivates us to develop and characterize different families of artificial base pairs in the future, thus generating a pool of candidates from which one can select according to the respective requirements.

## Conflict of interest

The authors declare no conflict of interest.

## Biographical Information

*Karin Betz studied Life Sciences at the University of Konstanz (Germany), where she obtained her PhD degree in 2014 under the supervision of Prof. Dr. Andreas Marx. Currently, she is senior scientist in the same group. Her research interests include structural studies on DNA polymerases and other enzymes that process or are regulated by nucleotide species*.

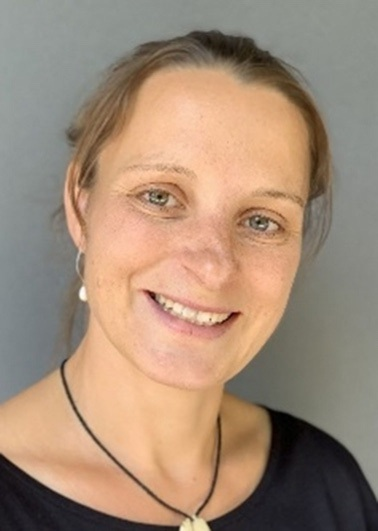

## Biographical Information

*Andreas Marx graduated from Bernd Giese's group at the University of Basel, Switzerland, in 1997. Afterwards, he joined Hisashi Yamamoto's group at Nagoya University, Japan, as a JSPS/EU postdoctoral fellow. At the end of 1999, he started his Habilitation with Michael Famulok at the University of Bonn and obtained the venia legendi in organic chemistry and biochemistry in 2003. In January 2004, he took up a position at the University of Konstanz (Germany). He is currently a member of the Heidelberg Academy of Sciences and Humanities and Study Section Chemistry at the German Science Foundation. In 2013, he was awarded an ERC Advanced Grant and in 2014 the Karl Heinz Beckurts Prize*.

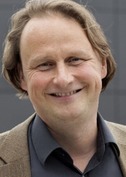

## References

[1] A. Rich , Horiz. Biochem. Biophys. 1962, 103–126.

[2] M. Kimoto , R. Yamashige , K.-i. Matsunaga , S. Yokoyama , I. Hirao , Nat. Biotechnol. 2013, 31, 453–457. 23563318 10.1038/nbt.2556

[3] K. Sefah , Z. Yang , K. M. Bradley , S. Hoshika , E. Jiménez , L. Zhang , G. Zhu , S. Shanker , F. Yu , D. Turek , W. Tan , S. A. Benner , Proc. Natl. Acad. Sci. USA 2014, 111, 1449–1454. 24379378 10.1073/pnas.1311778111 PMC3910645

[4] L. Zhang , Z. Yang , K. Sefah , K. M. Bradley , S. Hoshika , M.-J. Kim , H.-J. Kim , G. Zhu , E. Jiménez , S. Cansiz , I.-T. Teng , C. Champanhac , C. McLendon , C. Liu , W. Zhang , D. L. Gerloff , Z. Huang , W. Tan , S. A. Benner , J. Am. Chem. Soc. 2015, 137, 6734–6737. 25966323 10.1021/jacs.5b02251 PMC4500535

[5] L. Zhang , Z. Yang , T. L. Trinh , I.-T. Teng , S. Wang , K. M. Bradley , S. Hoshika , Q. Wu , S. Cansiz , D. J. Rowold , C. McLendon , M.-S. Kim , Y. Wu , C. Cui , Y. Liu , W. Hou , K. Stewart , S. Wan , C. Liu , S. A. Benner , W. Tan , Angew. Chem. Int. Ed. 2016, 55, 12372–12375; 10.1002/anie.201605058 PMC5554412 27601357

[6] E. Biondi , J. D. Lane , D. Das , S. Dasgupta , J. A. Piccirilli , S. Hoshika , K. M. Bradley , B. A. Krantz , S. A. Benner , Nucleic Acids Res. 2016, 44, 9565–9577. 27701076 10.1093/nar/gkw890 PMC5175368

[7] K. Futami , M. Kimoto , Y. W. S. Lim , I. Hirao , Mol. Ther. Nucleic Acids 2019, 14, 158–170. 30594072 10.1016/j.omtn.2018.11.011 PMC6307347

[8] J. D. Bain , C. Switzer , R. Chamberlin , S. A. Bennert , Nature 1992, 356, 537–539. 1560827 10.1038/356537a0

[9] I. Hirao , T. Ohtsuki , T. Fujiwara , T. Mitsui , T. Yokogawa , T. Okuni , H. Nakayama , K. Takio , T. Yabuki , T. Kigawa , K. Kodama , T. Yokogawa , K. Nishikawa , S. Yokoyama , Nat. Biotechnol. 2002, 20, 177–182. 11821864 10.1038/nbt0202-177

[10] Y. Zhang , J. L. Ptacin , E. C. Fischer , H. R. Aerni , C. E. Caffaro , K. San Jose , A. W. Feldman , C. R. Turner , F. E. Romesberg , Nature 2017, 551, 644–647. 29189780 10.1038/nature24659 PMC5796663

[11] Y. J. Seo , D. A. Malyshev , T. Lavergne , P. Ordoukhanian , F. E. Romesberg , J. Am. Chem. Soc. 2011, 133, 19878–19888. 21981600 10.1021/ja207907d PMC3988912

[12] L. Li , M. Degardin , T. Lavergne , D. A. Malyshev , K. Dhami , P. Ordoukhanian , F. E. Romesberg , J. Am. Chem. Soc. 2014, 136, 826–829. 24152106 10.1021/ja408814g PMC3979842

[13] J. Riedl , Y. Ding , A. M. Fleming , C. J. Burrows , Nat. Commun. 2015, 6, 8807. 26542210 10.1038/ncomms9807 PMC4667634

[14] Z. Yang , F. Chen , S. Chamberlin , S. Benner , Angew. Chem. Int. Ed. 2010, 49, 177–180; 10.1002/anie.200905173 PMC3155763 19946925

[15] L. G. Glushakova , A. Bradley , K. M. Bradley , B. W. Alto , S. Hoshika , D. Hutter , N. Sharma , Z. Yang , M. J. Kim , S. A. Benner , J. Virol. Methods 2015, 214, 60–74. 25680538 10.1016/j.jviromet.2015.01.003 PMC4485418

[16] K. K. Merritt , K. M. Bradley , D. Hutter , M. F. Matsuura , D. J. Rowold , S. A. Benner , Beilstein J. Org. Chem. 2014, 10, 2348–2360. 25383105 10.3762/bjoc.10.245 PMC4222377

[17] M. Kimoto , R. S. Cox III , I. Hirao , Expert Rev. Mol. Diagn. 2011, 11, 321–331. 21463241 10.1586/erm.11.5

[18] S. A. Benner , Acc. Chem. Res. 2004, 37, 784–797. 15491125 10.1021/ar040004z

[19] F. Wojciechowski , C. J. Leumann , Chem. Soc. Rev. 2011, 40, 5669–5679. 21431120 10.1039/c1cs15027h

[20] I. Hirao , M. Kimoto , Proc. Jpn. Acad. Ser. B 2012, 88, 345–367. 22850726 10.2183/pjab.88.345 PMC3422687

[21] I. Hirao , M. Kimoto , R. Yamashige , Acc. Chem. Res. 2012, 45, 2055–2065. 22263525 10.1021/ar200257x

[22] D. A. Malyshev , F. E. Romesberg , Angew. Chem. Int. Ed. 2015, 54, 11930–11944; 10.1002/anie.201502890 PMC4798003 26304162

[23] A. W. Feldman , F. E. Romesberg , Acc. Chem. Res. 2018, 51, 394–403. 29198111 10.1021/acs.accounts.7b00403 PMC5820176

[24] S. A. Benner , N. B. Karalkar , S. Hoshika , R. Laos , R. W. Shaw , M. Matsuura , D. Fajardo , P. Moussatche , Cold Spring Harbor Perspect. Biol. 2016, 8, a023770. 10.1101/cshperspect.a023770 PMC5088529 27663774

[25] K. H. Lee , K. Hamashima , M. Kimoto , I. Hirao , Curr. Opin. Biotechnol. 2018, 51, 8–15. 29049900 10.1016/j.copbio.2017.09.006

[26] E. Eremeeva , P. Herdewijn , Curr. Opin. Biotechnol. 2019, 57, 25–33. 30554069 10.1016/j.copbio.2018.12.001

[27] C. R. Geyer , T. R. Battersby , S. A. Benner , Structure 2003, 11, 1485–1498. 14656433 10.1016/j.str.2003.11.008

[28] Z. Yang , D. Hutter , P. Sheng , A. M. Sismour , S. A. Benner , Nucleic Acids Res. 2006, 34, 6095–6101. 17074747 10.1093/nar/gkl633 PMC1635279

[29] Z. Yang , A. M. Sismour , P. Sheng , N. L. Puskar , S. A. Benner , Nucleic Acids Res. 2007, 35, 4238–4249. 17576683 10.1093/nar/gkm395 PMC1934989

[30] Z. Yang , F. Chen , J. B. Alvarado , S. A. Benner , J. Am. Chem. Soc. 2011, 133, 15105–15112. 21842904 10.1021/ja204910n PMC3427765

[31] R. Laos , R. Shaw , N. A. Leal , E. Gaucher , S. Benner , Biochemistry 2013, 52, 5288–5294. 23815560 10.1021/bi400558c

[32] I. Singh , R. Laos , S. Hoshika , S. A. Benner , M. M. Georgiadis , Nucleic Acids Res. 2018, 46, 7977–7988. 29986111 10.1093/nar/gky552 PMC6125688

[33] L. F. Reichenbach , A. A. Sobri , N. R. Zaccai , C. Agnew , N. Burton , L. P. Eperon , S. de Ornellas , I. C. Eperon , R. L. Brady , G. A. Burley , Chem 2016, 1, 946–958.

[34] E. T. Kool , Biopolymers 1998, 48, 3–17. 9846123 10.1002/(SICI)1097-0282(1998)48:1<3::AID-BIP2>3.0.CO;2-7

[35] J. C. Morales , E. T. Kool , J. Am. Chem. Soc. 1999, 121, 2323–2324. 20852718 10.1021/ja983502+ PMC2939743

[36] R. Yamashige , M. Kimoto , Y. Takezawa , A. Sato , T. Mitsui , S. Yokoyama , I. Hirao , Nucleic Acids Res. 2012, 40, 2793–2806. 22121213 10.1093/nar/gkr1068 PMC3315302

[37] I. Okamoto , Y. Miyatake , M. Kimoto , I. Hirao , ACS Synth. Biol. 2016, 5, 1220–1230. 26814421 10.1021/acssynbio.5b00253

[38] M. Kimoto , R. Kawai , T. Mitsui , S. Yokoyama , I. Hirao , Nucleic Acids Res. 2009, 37, e14. 19073696 10.1093/nar/gkn956 PMC2632903

[39] D. A. Malyshev , K. Dhami , T. Lavergne , T. Chen , N. Dai , J. M. Foster , I. R. Corrêa , F. E. Romesberg , Nature 2014, 509, 385–388. 24805238 10.1038/nature13314 PMC4058825

[40] D. A. Malyshev , K. Dhami , H. T. Quach , T. Lavergne , P. Ordoukhanian , A. Torkamani , F. E. Romesberg , Proc. Natl. Acad. Sci. USA 2012, 109, 12005–12010. 22773812 10.1073/pnas.1205176109 PMC3409741

[41] Y. Zhang , B. M. Lamb , A. W. Feldman , A. X. Zhou , T. Lavergne , L. Li , F. E. Romesberg , Proc. Natl. Acad. Sci. 2017, 201616443. 10.1073/pnas.1616443114 PMC5307467 28115716

[42] V. T. Dien , M. Holcomb , A. W. Feldman , E. C. Fischer , T. J. Dwyer , F. E. Romesberg , J. Am. Chem. Soc. 2018, 140, 16115–16123. 30418780 10.1021/jacs.8b08416 PMC6373772

[43] A. W. Feldman , F. E. Romesberg , J. Am. Chem. Soc. 2017, 139, 11427–11433. 28796508 10.1021/jacs.7b03540 PMC5603228

[44] M. P. Ledbetter , R. J. Karadeema , F. E. Romesberg , J. Am. Chem. Soc. 2018, 140, 758–765. 29309130 10.1021/jacs.7b11488 PMC5793209

[45] J. Gao , H. Liu , E. T. Kool , J. Am. Chem. Soc. 2004, 126, 11826–11831. 15382917 10.1021/ja048499a

[46] H. Lu , A. T. Krueger , J. Gao , H. Liu , E. T. Kool , Org. Biomol. Chem. 2010, 8, 2704–2710. 20407680 10.1039/c002766a PMC5506547

[47] N. Minakawa , S. Ogata , M. Takahashi , A. Matsuda , J. Am. Chem. Soc. 2009, 131, 1644–1645. 19146369 10.1021/ja807391g

[48] P. Scharf , J. Müller , ChemPlusChem 2013, 78, 20–34.

[49] Y. Takezawa , M. Shionoya , Acc. Chem. Res. 2012, 45, 2066–2076. 22452649 10.1021/ar200313h

[50] B. Jash , J. Müller , Chem. Eur. J. 2017, 23, 17166–17178. 28833684 10.1002/chem.201703518

[51] S. Jahiruddin , A. Datta , J. Phys. Chem. B 2015, 119, 5839–5845. 25893481 10.1021/acs.jpcb.5b03293

[52] S. Jahiruddin , N. Mandal , A. Datta , ChemPhysChem 2018, 19, 67–74. 29139595 10.1002/cphc.201700997

[53] I. Negi , P. Kathuria , P. Sharma , S. D. Wetmore , Phys. Chem. Chem. Phys. 2017, 19, 16365–16374. 28657627 10.1039/c7cp02576a

[54] R. Galindo-Murillo , J. Barroso-Flores , Phys. Chem. Chem. Phys. 2017, 19, 10571–10580. 28394373 10.1039/c7cp01477e

[55] K. Betz , D. A. Malyshev , T. Lavergne , W. Welte , K. Diederichs , T. J. Dwyer , P. Ordoukhanian , F. E. Romesberg , A. Marx , Nat. Chem. Biol. 2012, 8, 612–614. 22660438 10.1038/nchembio.966 PMC3690913

[56] D. A. Malyshev , D. A. Pfaff , S. I. Ippoliti , G. T. Hwang , T. J. Dwyer , F. E. Romesberg , Chem. Eur. J. 2010, 16, 12650–12659. 20859962 10.1002/chem.201000959 PMC3332063

[57] S. Hoshika , I. Singh , C. Switzer , R. W. Molt , N. A. Leal , M.-J. Kim , M.-S. Kim , H.-J. Kim , M. M. Georgiadis , S. A. Benner , J. Am. Chem. Soc. 2018, 140, 11655–11660. 30148365 10.1021/jacs.8b05042

[58] C. Brotschi , C. J. Leumann , Angew. Chem. Int. Ed. 2003, 42, 1655–1658; 10.1002/anie.200250516 12698469

[59] C. Brotschi , G. Mathis , C. J. Leumann , Chem. Eur. J. 2005, 11, 1911–1923. 15685710 10.1002/chem.200400858

[60] Z. Johar , A. Zahn , C. J. Leumann , B. Jaun , Chem. Eur. J. 2008, 14, 1080–1086. 18038386 10.1002/chem.200701304

[61] S. Matsuda , A. M. Leconte , F. E. Romesberg , J. Am. Chem. Soc. 2007, 129, 5551–5557. 17411040 10.1021/ja068282b PMC2527036

[62] V. L. Malinovskii , D. Wenger , R. Häner , Chem. Soc. Rev. 2010, 39, 410–422. 20111767 10.1039/b910030j

[63] Y. N. Teo , E. T. Kool , Chem. Rev. 2012, 112, 4221–4245. 22424059 10.1021/cr100351g PMC3387364

[64] T. Mitsui , A. Kitamura , M. Kimoto , T. To , A. Sato , I. Hirao , S. Yokoyama , J. Am. Chem. Soc. 2003, 125, 5298–5307. 12720441 10.1021/ja028806h

[65] H. Echols , M. F. Goodman , Annu. Rev. Biochem. 1991, 60, 477–511. 1883202 10.1146/annurev.bi.60.070191.002401

[66] T. A. Kunkel , J. Biol. Chem. 2004, 279, 16895–16898. 14988392 10.1074/jbc.R400006200

[67] P. J. Rothwell , G. Waksman , Adv. Protein Chem. 2005, 71, 401–440. 16230118 10.1016/S0065-3233(04)71011-6

[68] E. Y. Wu , L. S. Beese , J. Biol. Chem. 2011, 286, 19758–19767. 21454515 10.1074/jbc.M110.191130 PMC3103354

[69] K. Betz , D. A. Malyshev , T. Lavergne , W. Welte , K. Diederichs , F. E. Romesberg , A. Marx , J. Am. Chem. Soc. 2013, 135, 18637–18643. 24283923 10.1021/ja409609j PMC3982147

[70] K. Betz , M. Kimoto , K. Diederichs , I. Hirao , A. Marx , Angew. Chem. Int. Ed. 2017, 56, 12000–12003; 10.1002/anie.201704190 28594080

[71] E. T. Kool , Annu. Rev. Biochem. 2002, 71, 191–219. 12045095 10.1146/annurev.biochem.71.110601.135453

[72] K. Bergen , A.-L. Steck , S. Strütt , A. Baccaro , W. Welte , K. Diederichs , A. Marx , J. Am. Chem. Soc. 2012, 134, 11840–11843. 22475415 10.1021/ja3017889

[73] S. Obeid , A. Baccaro , W. Welte , K. Diederichs , A. Marx , Proc. Natl. Acad. Sci. USA 2010, 107, 21327–21331. 21123743 10.1073/pnas.1013804107 PMC3003043

[74] A. Hottin , K. Betz , K. Diederichs , A. Marx , Chem. Eur. J. 2017, 23, 2109–2118. 27901305 10.1002/chem.201604515

[75] X.-J. Lu , W. K. Olson , Nat. Protoc. 2008, 3, 1213–1227. 18600227 10.1038/nprot.2008.104 PMC3065354

[76] P. Emsley , B. Lohkamp , W. G. Scott , K. Cowtan , Acta Crystallogr. Sect. D: Biol. Crystallogr. 2010, 66, 486–501. 20383002 10.1107/S0907444910007493 PMC2852313

[77] S. Obeid , N. Blatter , R. Kranaster , A. Schnur , K. Diederichs , W. Welte , A. Marx , EMBO J. 2010, 29, 1738–1747. 20400942 10.1038/emboj.2010.64 PMC2876968

[78] S. Obeid , W. Welte , K. Diederichs , A. Marx , J. Biol. Chem. 2012, 287, 14099–14108. 22318723 10.1074/jbc.M111.334904 PMC3340134

[79] Y. Santoso , C. M. Joyce , O. Potapova , L. Le Reste , J. Hohlbein , J. P. Torella , N. D. F. Grindley , A. N. Kapanidis , Proc. Natl. Acad. Sci. USA 2010, 107, 715–720. 20080740 10.1073/pnas.0910909107 PMC2818957

[80] S. Y. Berezhna , J. P. Gill , R. Lamichhane , D. P. Millar , J. Am. Chem. Soc. 2012, 134, 11261–11268. 22650319 10.1021/ja3038273 PMC3448555

[81] J. Hohlbein , L. Aigrain , T. D. Craggs , O. Bermek , O. Potapova , P. Shoolizadeh , N. D. F. Grindley , C. M. Joyce , A. N. Kapanidis , Nat. Commun. 2013, 4, 2131. 23831915 10.1038/ncomms3131 PMC3715850

[82] P. J. Rothwell , W. J. Allen , E. Sisamakis , S. Kalinin , S. Felekyan , J. Widengren , G. Waksman , C. A. M. Seidel , J. Biol. Chem. 2013, 288, 13575–13591. 23525110 10.1074/jbc.M112.432690 PMC3650393

[83] J. Hohlbein , A. Kapanidis , Probing the Conformational Landscape of DNA Polymerases Using Diffusion-Based Single-Molecule FRET in Single-Molecule Enzymology: Fluorescence-Based and High-Throughput Methods, Vol. 581 of Methods in Enzymology (Eds.: M. Spies, Y. R. Chemla), Academic Press, Cambridge, MA, 2016, Chapter 12, pp. 353–378. 10.1016/bs.mie.2016.08.023 27793286

[84] T. Lavergne , D. A. Malyshev , F. E. Romesberg , Chem. Eur. J. 2012, 18, 1231–1239. 22190386 10.1002/chem.201102066 PMC3693734

[85] K. Betz, Dissertation, University of Konstanz, **2014**.

[86] Y. J. Seo , G. T. Hwang , P. Ordoukhanian , F. E. Romesberg , J. Am. Chem. Soc. 2009, 131, 3246–3252. 19256568 10.1021/ja807853m PMC2901498

[87] A. A. Golosov , J. J. Warren , L. S. Beese , M. Karplus , Structure 2010, 18, 83–93. 20152155 10.1016/j.str.2009.10.014 PMC3325112

[88] M. M. Georgiadis , I. Singh , W. F. Kellett , S. Hoshika , S. A. Benner , N. G. J. Richards , J. Am. Chem. Soc. 2015, 137, 6947–6955. 25961938 10.1021/jacs.5b03482 PMC4633024

[89] R. W. J. Molt , M. M. Georgiadis , N. G. J. Richards , Nucleic Acids Res. 2017, 45, 3643–3653. 28334863 10.1093/nar/gkx144 PMC5397145

[90] S. Hoshika , N. A. Leal , M.-J. Kim , M.-S. Kim , N. B. Karalkar , H.-J. Kim , A. M. Bates , N. E. Watkins , H. A. SantaLucia , A. J. Meyer , S. DasGupta , J. A. Piccirilli , A. D. Ellington , J. SantaLucia , M. M. Georgiadis , S. A. Benner , Science 2019, 363, 884–887. 30792304 10.1126/science.aat0971 PMC6413494

